# STAT3 expression by myeloid cells is detrimental for the T- cell-mediated control of infection with *Mycobacterium tuberculosis*

**DOI:** 10.1371/journal.ppat.1006809

**Published:** 2018-01-16

**Authors:** Yu Gao, Juan Ignacio Basile, Cajsa Classon, Dolores Gavier-Widen, Akihiko Yoshimura, Berit Carow, Martin E. Rottenberg

**Affiliations:** 1 Department of Microbiology, Tumor and Cell Biology, Karolinska Institutet, Stockholm, Sweden; 2 Department of Pathology and Wild Life Diseases, Swedish National Veterinary Institute, Uppsala, Sweden; 3 Department of Microbiology and Immunology, Keio University School of Medicine, Tokyo, Japan; University of Massachusetts Medical School, UNITED STATES

## Abstract

STAT3 is a master regulator of the immune responses. Here we show that *M*. *tuberculosis*-infected *stat3*^*fl/fl*^ *lysm cre* mice, defective for STAT3 in myeloid cells, contained lower bacterial load in lungs and spleens, reduced granuloma extension but higher levels of pulmonary neutrophils. STAT3-deficient macrophages showed no improved control of intracellular mycobacterial growth. Instead, protection associated to elevated ability of *stat3*^*fl/fl*^ *lysm cre* antigen-presenting cells (APCs) to release IL-6 and IL-23 and to stimulate IL-17 secretion by mycobacteria-specific T cells. The increased IL-17 secretion accounted for the improved control of infection since neutralization of IL-17 receptor A in *stat3*^*fl/fl*^ *lysm cre* mice hampered bacterial control. APCs lacking SOCS3, which inhibits STAT3 activation via several cytokine receptors, were poor inducers of priming and of the IL-17 production by mycobacteria-specific T cells. In agreement, *socs3*^*fl/fl*^ *cd11c cre* mice deficient of SOCS3 in DCs showed increased susceptibility to *M*. *tuberculosis* infection. While STAT3 in APCs hampered IL-17 responses, STAT3 in mycobacteria-specific T cells was critical for IL-17 secretion, while SOCS3 in T cells impeded IL-17 secretion. Altogether, STAT3 signalling in myeloid cells is deleterious in the control of infection with *M*. *tuberculosis*.

## Author summary

We studied the role of STAT3, a major regulator of immunity, in the control of the infection with *M*. *tuberculosis*. *Stat3*^*fl/fl*^ *lysm cre* mice, deficient in STAT3 in myeloid cells, showed lower bacterial levels in organs and reduced extension of lung granulomas after infection with *M*. *tuberculosis*. STAT3-deficient APCs stimulated with innate receptor agonists released high levels of IL-6 and IL-23, and promoted IL-17 production by mycobacteria-specific CD4+ T cells. Increased IL-17 levels accounted for the increased resistance to *M*. *tuberculosi*s of the STAT3-deficient mice. Instead, *stat3*^*fl/fl*^ *lysm cre* macrophages showed no improved control of mycobacterial growth. SOCS3 is a negative regulator of STAT3 activation. The ability of s*ocs3*^*fl/fl*^ *lysm cre* APCs to secrete IL-6 and IL-23 and to stimulate IL-17 production by antigen-specific T cells was reduced. In agreement, mice lacking SOCS3 in DCs showed increased susceptibility to *M*. *tuberculosis* infection. Different to a role in myeloid cells, STAT3 expression by mycobacteria-specific T cells was required for IL-17 secretion while SOCS3 in T cells hampered IL-17 production. Therefore, despite STAT3 expression in T cells is required for Th17 differentiation, STAT3 in APCs hampers secretion of Th17 promoting cytokines and the secretion of IL-17 by mycobacteria-specific T cells and reduces the resistance of mice to infection with *M*. *tuberculosis*.

## Introduction

Tuberculosis (TB), caused by infection with *Mycobacterium tuberculosis*, remains a leading public health problem worldwide. TB causes 9 million new cases and 1.5 million deaths each year [1]. However, host factors determining the outcome of infection are not completely understood.

A host counters mycobacterial infections primarily via T_H_1 immune responses that involve cellular effector mechanisms such as macrophage activation [2, 3]. IL-12 secreted by APCs is crucial for the differentiation and maintenance of IFN-γ-secreting antigen-specific T_H_1 cells [4, 5] and both IL-12 and IFN-γ mediate mycobacterial control in mice and man [6–9].

The transcription factor STAT3 is a central regulator of immunity, mediating inflammatory but also anti-inflammatory responses [10, 11]. The functions of STAT3 are pleiotropic. STAT3 is activated by phosphorylation in response to cytokines of the IFN-receptor family (such as IL-10) and by some members of the IL-2 receptor family that uses the common γ chain receptor or after stimulation of several receptor tyrosine kinases (EGF, CSF-1, and PDGF). Additionally, STAT3 is activated by the common signal transducing molecule gp130 utilized by the IL-6 receptor family [12], and in response to G-CSF and leptin as their receptors are homologous to gp130.

STAT3 is critical for defense against bacterial and fungal infections. Low IL-17 secreting T-cell proportions were reported in patients bearing STAT3 mutations. These patients were prone to chronic candidiasis and staphylococcal diseases [13]. Chronic candidiasis is frequently present in patients deficient in IL-17 receptor A [14]. STAT3 deficient patients may also display impaired immunity against chronic viral infections [15, 16].

In mice, knockout of STAT3 is lethal, so *in vivo* studies on STAT3 functions have been performed using conditional knock out mice. S*tat3*^*fl/fl*^ *lysm cre* mice, deficient in STAT3 in myeloid cells, display enhanced susceptibility to endotoxic shock and develop chronic enterocolitis with age [17]. The phenotype of these animals is similar to IL-10^-/-^ mice, including increased expression of TNF and other inflammatory cytokines, since IL-10 suppresses induction of TNF-α via STAT3 [18]. Recently, STAT3 was shown to favour intracellular growth of *M*. *tuberculosis* in human macrophages [19]. Moreover, the presence of pSTAT3+ monocytes associated with the progression of the disease in *M*. *tuberculosis* infected non-human primates [20].

We have previously analysed the role of SOCS3, a molecule that inhibits STAT3 activation after triggering of several cytokine and growth factor receptors, and found that mice devoid in SOCS3 in myeloid or lymphoid cells showed increased susceptibility to *M*. *tuberculosis* [21].

The role of STAT3 during infection with *M*. *tuberculosis* **in vivo** is still unknown. We here examine the role of STAT3 in *M*. *tuberculosis* by using *stat3*^*fl/fl*^ *lysm cre* mice. We highlight that STAT3 expression in APCs inhibits T_H_17 associated responses resulting in an increased susceptibility to infection with *M*. *tuberculosis*.

## Results

### Stat3^fl/fl^ lysm cre mice are resistant to infection with *M*. *tuberculosis*

**Fig 1:**
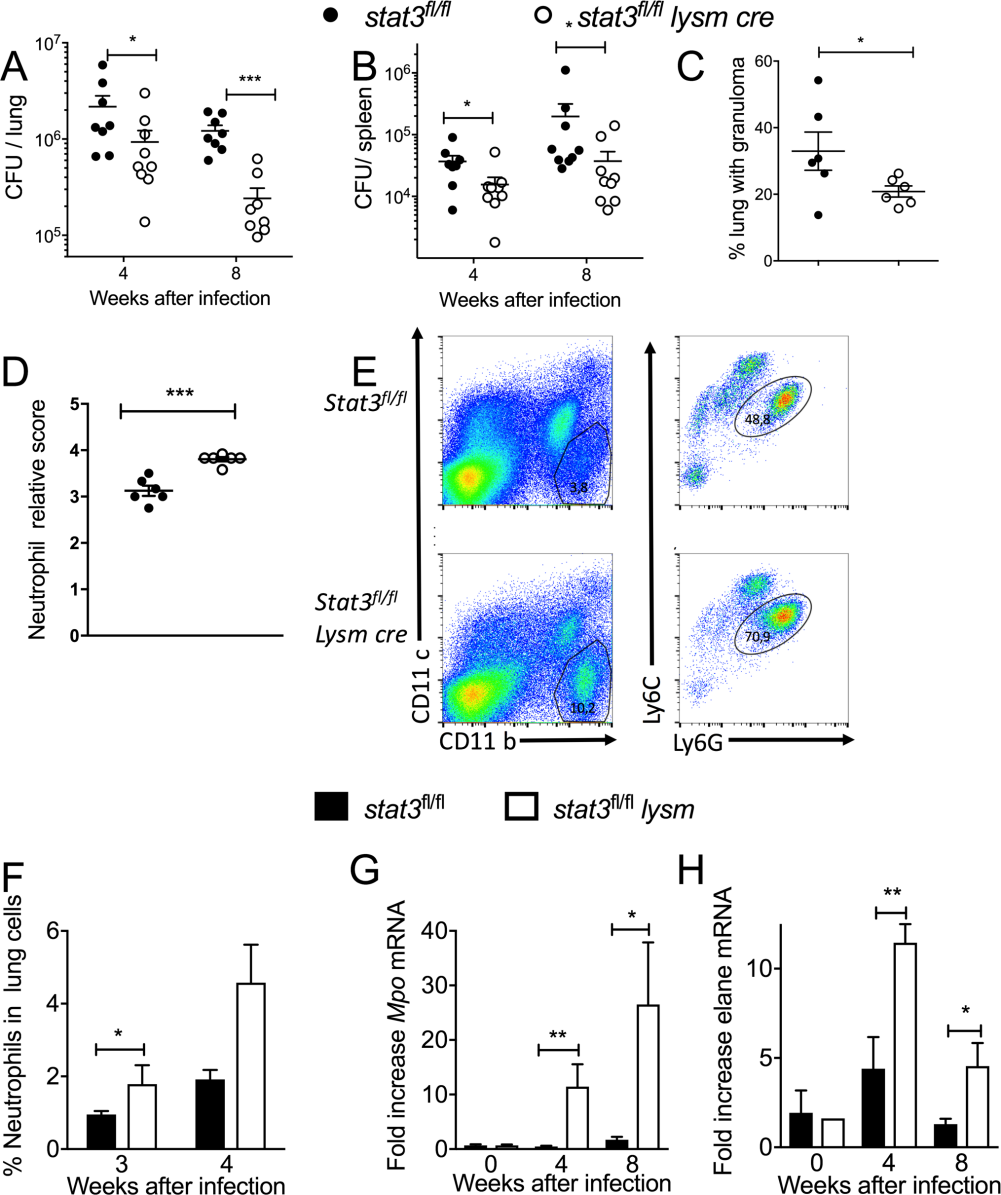
*Stat3*^*fl/fl*^ *lysm cre* mice are resistant to infection with *M*. *tuberculosis*. *Stat3*^*fl/fl*^ *lysm cre* and *stat3*^*fl/fl*^ littermate controls were sacrificed at indicated time points after aerosol infection with *M*. *tuberculosis* and colony forming units (CFU) per lung (A) and spleen (B) were assessed. The CFU per organ of individual mice and the median per group at the indicated time points after infection are depicted. For each time point 8–10 control and 8–10 mutant mice were infected simultaneously. We performed separated experiments for 4 and 8 weeks post infection. Only one representative of the two experiments for 4 as well as 8 weeks post infection is depicted. Differences in CFU are significant (*p<0.05, **p<0.01, ***p<0.001 Mann Whitney U test). Histopathological scoring of hematoxylin-eosin stained paraffin lung sections from *stat3*^*fl/fl*^ *lysm cre* and *stat3*^*fl/fl*^ mice measured 4 and 8 weeks after infection with *M*. *tuberculosis*. The mean ± SEM % lung area with granulomas (C) and the relative neutrophil density score (D) are depicted. Differences are significant (*p<0.05, ***p<0.001 Student t test). A representative dot plot (E) and the frequency (F) of CD11b^+^CD11c^-^Ly6C^dim^ Ly6G^+^ neutrophils in lungs *stat3*^*fl/fl*^ *lysm cre* and *stat3*^*fl/fl*^ mice 3 and 4 weeks after infection with *M*. *tuberculosis* are shown. Differences between 4 mice at each time point are significant (*p<0.05 Student’s t test). Total RNA was extracted from the lungs of *stat3*^*fl/fl*^ *lysm cre* and *stat3*^*fl/fl*^ mice at the indicated time points after infection with *M*. *tuberculosis*. The relative concentration of neutrophil elastase (*elane*) (G) and myeloperoxidase (*mpo)* transcripts (H) in relation to *hprt* mRNA levels in the same sample was determined by real time PCR. The mean fold induction of these transcripts ± SEM is depicted. Differences with control mice are significant (*p<0.05, **p<0.01 Student t-test).

First, the role of STAT3 expression in myeloid cells in the control of infection with *M*. *tuberculosis* was examined using *stat3*^*fl/fl*^ *lysm cre* mice. Lungs and spleens from *stat3*^*fl/fl*^ *lysm cre* mice after 4 and 8 weeks of infection showed significantly lower *M*. *tuberculosis* burden than *stat3*^*fl/fl*^ littermates (Fig 1A and 1B). A smaller area of the lung parenchyma of *stat3*^*fl/fl*^ *lysm cre* mice was occupied by granulomas when compared to control lungs 4 but not at 8 weeks after infection (Fig 1C).

The density of granulocytes in the lung parenchyma was determined either by H&E staining of sections (Fig 1D) or by labelling of CD11b^+^CD11c^-^Ly6C^dim^Ly6G^+^ neutrophils (Fig 1E and 1F) in lung suspensions from stat3^*fl/fl*^ *lysm cre* and stat3^*fl/fl*^ mice 3 and 4 weeks after *M*. *tuberculosis* infection. The neutrophil density (Fig 1D–1F) and the levels of neutrophil myeloperoxidase *(mpo)* and elastase *(elane)* mRNAs (Fig 1G and 1H) were also higher in lungs from *stat3*^*fl/fl*^ *lysm cre* at mice 4 and 8 but not at 14 weeks after infection *with M*. *tuberculosis*- compared to controls (Fig 1D–1H and S1A–S1C Fig).

### Stat3-deficient and control BMM show similar control of the growth of intracellular *M*. *tuberculosis*

**Fig 2:**
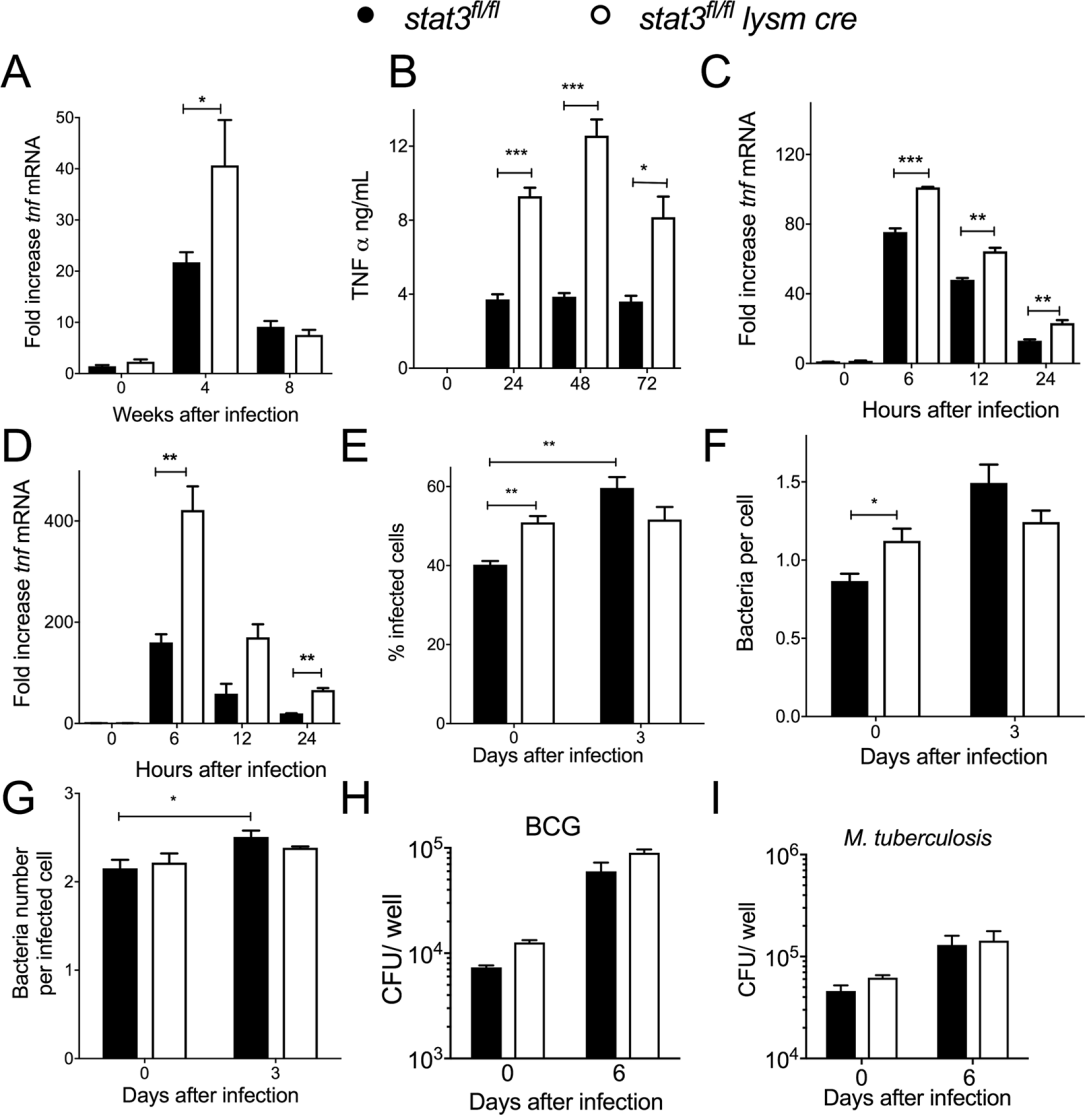
Stat3 deficient and control BMM show similar control of the intracellular growth of *M*. *tuberculosis*. The levels of *tnf* mRNA in the lungs from *stat3*^*fl/fl*^ *lysm cre* and *stat3*^*fl/fl*^ mice at the indicated time points after infection with *M*. *tuberculosis* (A), and in *stat3*^*fl/fl*^ *lysm cre* and *stat3*^*fl/fl*^ BMM incubated with *M*. *tuberculosis* (C) or BCG (D) were determined by real time PCR. The mean fold increase of mRNA level ± SEM in 8 mice per group (A) or in triplicate independent cultures per condition compared to non-infected cultures (C, D) of one of two independent experiments is depicted (*p<0.05, **p<0.01, ***p<0.001 Student t test). The mean concentration of TNF in the supernatants of *stat3*^*fl/fl*^ *lysm cre* and *stat3*^*fl/fl*^ BMM at different times after *M*. *tuberculosis* infection were measured by ELISA (B). The mean % of infected BMM (E), the bacteria number per BMM (F) and the bacteria per infected BMM (G) ± SEM at 0 and 3 days after infection of *stat3*^*fl/fl*^ *lysm cre* and *stat3*^*fl/fl*^ BMM were determined after staining with auramin-rhodamin T for mycobacterial and DAPI for nuclei (E-G). One out of 2 independent experiments performed is depicted. Bacterial CFU were determined in *stat3*^*fl/fl*^ *lysm cre* and *stat3*^*fl/fl*^ BMM after infection with BCG (H) or *M*. *tuberculosis* (I) at a MOI of 5:1. The mean CFU ± SEM from triplicate cell cultures is shown. Three independent experiments for each panel were performed.

Activated STAT3 hampers TNF expression [22, 23]. Lungs from *stat3*^*fl/fl*^ *lysm cre* mice infected with *M*. *tuberculosis* (Fig 2A) as well as BMM infected with *M*. *tuberculosis* or BCG (Fig 2B–2D) showed higher TNF protein and mRNA levels than controls. Since TNF has been shown to mediate *M*. *tuberculosis* control in macrophages [24], we speculated that *stat3*^*fl/fl*^ *lysm cre* macrophages could display a better control of intracellular mycobacteria.

A higher frequency of *stat3*^*fl/fl*^ *lysm cre* BMMs were infected when measured 4 h after co-incubation with the *M*. *tuberculosis*, although the number of bacteria per infected cell was similar (Fig 2E–2G). Three days after infection *M*. *tuberculosis* infected mutant and WT BMM showed similar numbers of infected cells and bacteria per total or infected cell (Fig 2E–2G). S*tat3*^*fl/fl*^ *lysm cre* BMM showed no improved control of *M*. *tuberculosis* or BCG growth **in vitro** 6 days after infection as measured by CFU in lysates (Fig 2H and 2I). Altogether, we observed no indication of an improved bacterial growth control or reduced bacterial uptake in stat3^*fl/fl*^ *lysm cre* BMM.

### Role of STAT3 and SOCS3 in APCs in the regulation of T cell priming

**Fig 3:**
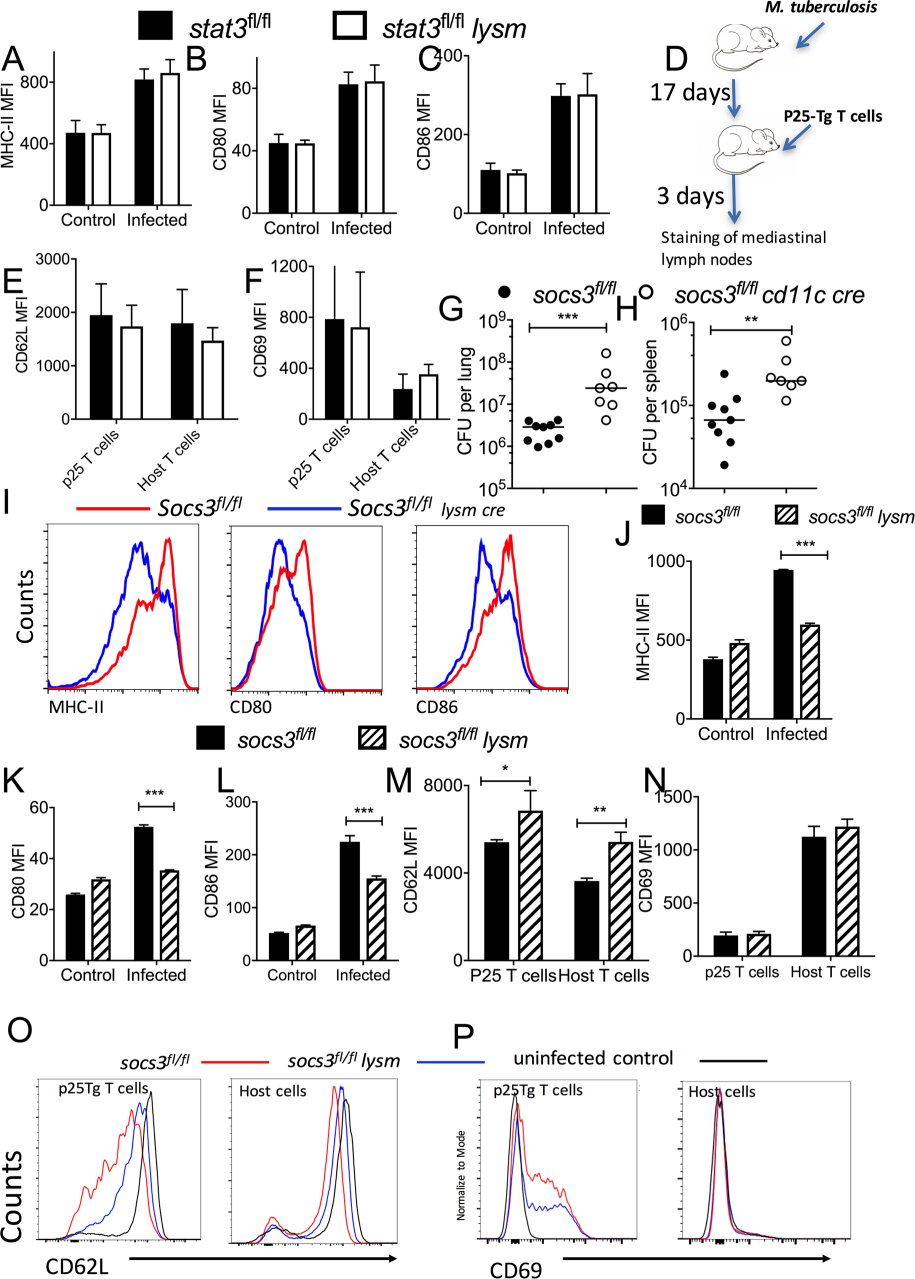
Role of STAT3 and SOCS3 in APCs in the regulation of T cell priming. The median fluorescent intensity (MFI) of MHCII, CD80 and CD86 on CD11c^+^ *stat3*^*fl/fl*^ *lysm cre* or *stat3*^*fl/fl*^ (A-C) BMDCs was determined by FACS analysis 24 h after infection with BCG or non-infected controls. The mean MFI ± SEM are depicted. Experimental scheme of *M*. *tuberculosis* infection followed 17 days after by transfer of 3. 10^6^ *p25-tg* naïve T cells. Mice were sacrificed 3 days later (D). The mean MFI of CD62L (E) and CD69 (F) ± SEM on MLN CD4^+^ *p25-tg* or recipient T cells from either *stat3*^*fl/fl*^ lysm cre or *stat3*^*fl/fl*^ *M*. *tuberculosis-*infected mice. *Socs3*^*fl/fl*^ *cd11c cre and socs3*^*fl/fl*^ littermate controls were sacrificed 4 weeks after aerosol infection with *M*. *tuberculosis* and colony forming units (CFU) per lung (G) and spleen (H) were assessed. The CFU per organ of individual mice and the median per group at the indicated time points after infection are depicted. Differences in CFU are significant (**p<0.01, ***p<0.001 Mann Whitney U test). Representative FACS histograms and the mean MFI ± SEM of CD80, CD86 and MHC-II on *socs3*^*fl/fl*^ *lysm cre* and *socs3*^*fl/fl*^ BMDCs infected or not with BCG for 24 hs (I-L). Differences with *socs3*^*fl/fl*^ BMDCs (n = 4 cultures per group) are significant (*p<0.05, ***p<0.001 Student *t* test). Representative FACS histograms and mean MFI levels of CD62L (M, O) and CD69 (N, P) ± SEM on *p25-tg* and host CD4^+^ MLN T cells before or 21 days after infection with *M*. *tuberculosis*. Differences with *socs3*^*fl/fl*^ BMDCs are significant (*p<0.05 and **p<0.01 Student t test).

Several cytokines controlled by STAT3 are potent regulators of the expression of co-stimulatory molecules on APCs. Therefore, we studied whether STAT3 played a role in regulation of T cell priming. As expected, the density of co-stimulatory molecules CD80 and CD86 as well as MHC-II levels increased on BMDCs after mycobacterial stimulation. The expression of MHCII, CD80 and CD86 in either control or *stat3*^*fl/fl*^ *lysm cre* BMDCs before or after mycobacterial stimulation was similar (Fig 3A–3C). To investigate if the expression of STAT3 by myeloid cells could modulate T cell priming during infection with *M*. *tuberculosis* T cell receptor transgenic T cells specific for the immunodominant mycobacterial Ag85B_240-254_ peptide (*p25-tg*) cells were inoculated i.v. into *stat3*^*fl/fl*^ *lysm cre* or *stat3*^*fl/fl*^ mice 17 days after infection with *M*. *tuberculosis* (Fig 3D). Three days after transfer, the expression of CD69 (which increases after T cell receptor triggering) and CD62L (the L-selectin ligand that hampers T cells to traffic to the periphery) was measured on *p25-tg* T cells and host T cells from the mediastinal lymph nodes (MLN). The expression of CD69 was increased and the expression of CD62L was reduced in *p25-tg* T cells from MLN of infected mice when compared to uninfected control mice. Similar levels of the CD69 and CD62L were expressed by *p25-tg* or host T cells from *stat3*^*/fl*^ *lysm cre* or stat3^*fl/fl*^ infected mice (Fig 3E and 3F).

SOCS3 inhibits STAT3 activation by different cytokine receptors, e.g. those of the IL-6 receptor family [10]. In accordance with the results obtained with socs3^*fl/fl*^ *lysm cre* mice [21], *socs3*^*fl/fl*^ *cd11c cre* mice showed higher bacterial levels in lungs and spleens after infection with *M*. *tuberculosis* than control animals (Fig 3G and 3H). The *cd11c cre* transgene has been shown to be expressed in the majority of conventional and plasmacytoid DCs [25]

Mycobacteria-stimulated BMDC from *socs3*^*fl/fl*^ *lysm cre* showed lower levels of MHCII, CD80 and CD86 than control cells (Fig 3I–3L). When T cell priming **in vivo** was studied by transfering *p25-tg* naïve T cells into *M*. *tuberculosis* infected animals, the density of CD62L on donor *p25-tg* T cells and on host MLN T cells was lower in WT mice as compared to *socs3*^*fl/fl*^ *lysm cre* mice recipients (Fig 3M and 3O). The *p25-tg* T cells in the MLN of *socs3*^*fl/fl*^ *lysm cre* mice also showed lower surface density of CD69 as compared to those from WT-infected mice (Fig 3N and 3P). However, the expression of CD69 in host MLN T cells from *M*. *tuberculosis*-infected WT and *socs3*^*fl/fl*^ *lysm cre* mice was similar (Fig 3P).

Thus, deficiency of SOCS3 but not STAT3 in APCs regulates T cell priming during *M*. *tuberculosis* infection **in vivo**.

### STAT3 in myeloid cells impairs IFN-γ secretion by mycobacteria-specific T cells

**Fig 4:**
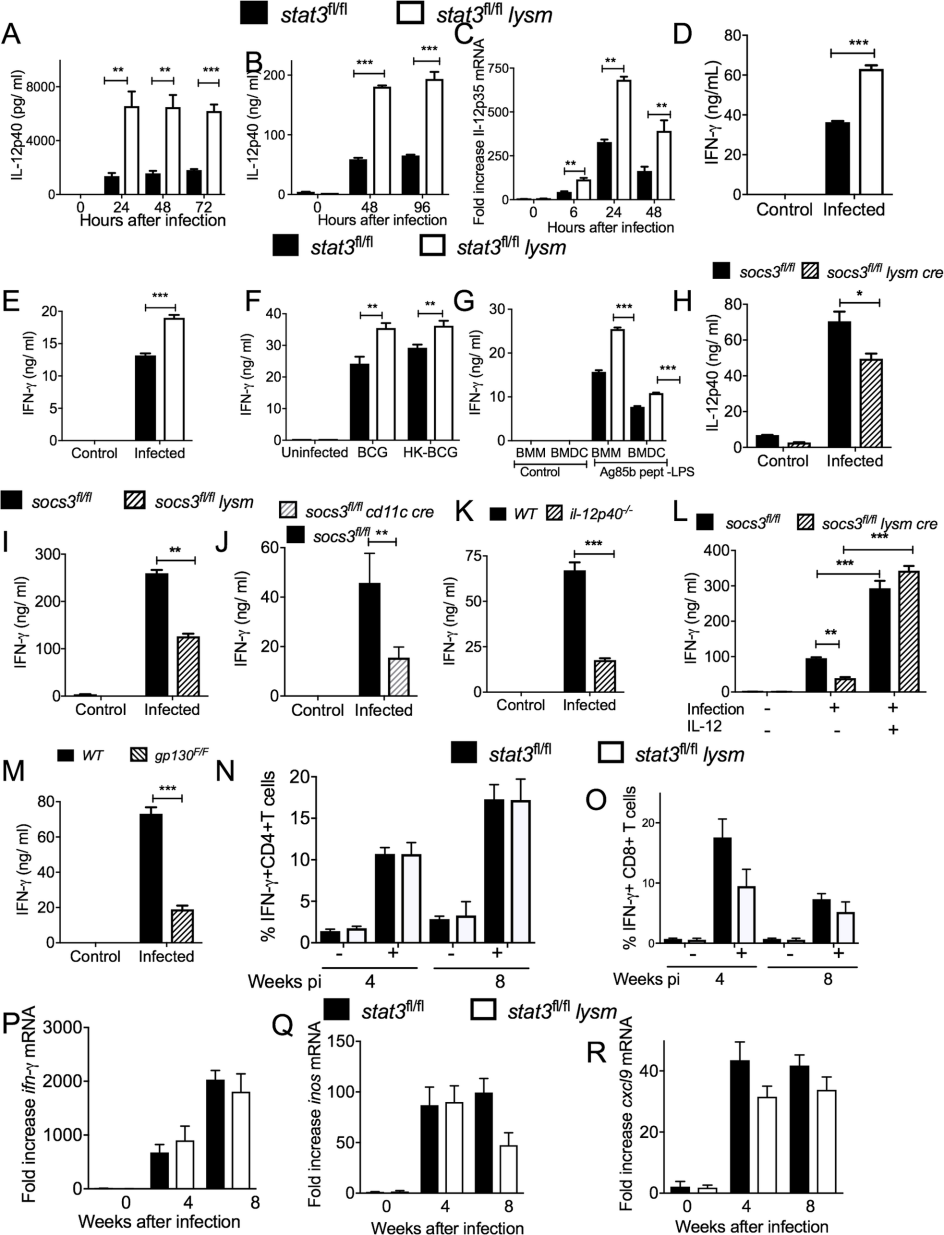
STAT3 in myeloid cells impairs IFN-γ secretion by mycobacteria-specific T cells in vitro. The concentration of IL-12 p40 in supernatants from mycobacteria-infected *stat3*^*fl/fl*^ *lysm cre* and *stat3*^*fl/fl*^ BMDCs (A) or BMM (B) at different times after incubation were determined by ELISA. The mean IL-12p40 ± SEM pg/ ml from triplicate cultures is depicted. Differences with *stat3*^*fl/fl*^ BMDCs are significant (**p<0.01, ***p<0.001 Student t test). Total RNA was extracted from *stat3*^*fl/fl*^ *lysm cre* and *stat3*^*fl/fl*^ BMDC cultures 24 h after *M*. *tuberculosis* infection. The mean *Il-12p35* mRNA levels ± SEM levels measured by real time PCR are depicted (C) (**p<0.01 Student t test). *Stat3*^*fl/fl*^ *lysm cre* and *stat3*^*fl/fl*^ BMDC were infected with either BCG (D), *M*. *tuberculosis* (E) or stimulated with heat killed BCG (F) or with LPS and peptide 25 of Ag85b (G) washed and incubated 6 h after with *p25-tg* CD4^+^ naïve T cells (at a ratio of 4:1 BMDC) (G). The concentration of IFN-γ in the culture supernatants was measured by ELISA 72h after co-incubation. The mean IFN-γ ± SEM from triplicate cultures is depicted (***p<0.001 Student t test). The concentration of IL-12p40 in supernatants from mycobacteria-infected *socs3*^*fl/fl*^ *lysm cre* and *socs3*^*fl/fl*^ BMDCs was determined by ELISA (H). The mean IL-12p40 ± SEM ng/ ml from triplicate cultures is depicted (**p<0.01 and ***p<0.001 Student t test). *Socs3*^*fl/fl*^ *lysm cre* (I), socs3^*fl/fl*^ *cd11 cre* (J) and *socs3*^*fl/fl*^ BMDC were infected with BCG and incubated 6 h after with *p25-tg* T cells. The concentration of IFN-γ in the supernatants was measured by ELISA 72h after co-incubation. The mean IFN-γ ± SEM from triplicate cultures is depicted (**p<0.01 Student t test). *Il12p40*^*-/-*^ (K), *gp130*^*F/F*^ (M) and WT BMDC were infected with BCG and co-incubated with *p25-tg* T cells as described. Mycobacterial-infected *socs3*^*fl/fl*^ *lysm cre* and *socs3*^*fl/fl*.^ were cultured in presence of recombinant IL-12p70 or left untreated (K) and co-incubated with p25Tg-T cells (L). The mean IFN-γ ± SEM in supernatants from triplicate cultures was measured by ELISA (**p<0.01, ***p<0.001 Student t test). The frequency of IFN-γ-secreting cells in PPD-stimulated pulmonary T cells from *stat3*^*fl/fl*^ *lysm cre* and *stat3*^*fl/fl*^ mice 4 and 8 weeks after infection with *M*. *tuberculosis* was analysed by ICS (N, O). The mean frequency of IFN-γ-secreting within CD4+ cells (N) and CD8+ (O) ± SEM is displayed (n = 5 per group). The levels of *ifng* (P), *inos* (Q)and *cxcl9* (R) mRNA in the lungs of *stat3*^*fl/fl*^ *lysm cre* and *stat3*^*fl/fl*^ mice before and at the indicated time points after aerosol infection with *M*. *tuberculosis* were determined by real time PCR.

IFN-γ is required for protection against *M*. *tuberculosis* [2, 3]. STAT3 has been shown to inhibit the transcription of IL-12, a potent inducer of IFN-γ secretion by T cells [23]. We then analysed if the increased resistance to *M*. *tuberculosis* of *stat3*^*fl/fl*^ *lysm cre* mice is associated with higher IFN-γ secretion by T cells. Higher levels of IL-12p40 (the α-chain of IL-12 and IL-23) in supernatants and *il12p40* mRNA in cell lysates of BCG-infected *stat3*^*fl/fl*^ *lysm cre* BMM or BMDC compared to controls were measured (Fig 4A and 4B). The *il12p35* mRNA coding for the β-chain of the IL-12 heterodimer was also expressed in higher amounts by *M*. *tuberculosis-* or BCG-stimulated *stat3*^*fl/fl*^ *lysm cre* BMDC compared to controls (Fig 4C).

Thus, whether STAT3-deficient APCs are better stimulators of IFN-γ secretion by mycobacteria-specific T cells than WT APCs was investigated. To test this hypothesis, *p25-tg* T cells were incubated with either BCG- or *M*. *tuberculosis*-infected *stat3*^*fl/fl*^ *lysm cre* or *stat3*^*fl/fl*^ BMDCs and the IFN-γ titers in the supernatants measured. IFN-γ levels in supernatants were elevated compared to those incubated with WT APCs (Fig 4D and 4E). Supernatants from cultures of *p25-tg* T cells incubated with heat-killed BCG-stimulated *stat3*^*fl/fl*^ BMDC or BMM also contained higher levels of IFN-γ than those using control APCs (Fig 4F), indicating that infection is not required for such responses. In line with this, IFN-γ levels were higher in supernatants from *p25-tg* T cells co-incubated with *stat3*^*fl/fl*^ *lysm cre* BMDC or BMM stimulated with oligopeptide p25 (amino acids 240–254) from Ag85b, a major immunodominant H2^b^ epitope [26] recognized by the *p25-tg* T cells, in presence of LPS (Fig 4G).

Confirming previous results [21, 27], *socs3*^*fl/fl*^ *lysm cre* BMDC showed diminished IL-12 secretion after mycobacterial stimulation (Fig 4H). Furthermore, IFN-γ secretion by *p25-tg* T-cells incubated with mycobacteria-infected *socs3*^*fl/fl*^ *lysm cre* or socs3 ^*fl/fl*^ *cd11 cre* BMDCs was reduced (Fig 4I and 4J).

In line with these results, mycobacteria-infected *il12p40*^-/-^ BMDCs showed reduced ability to trigger IFN-γ secretion by *p25-tg* T cells than controls (Fig 4K). Moreover, the addition of rec IL-12 restored the capacity of *socs3*^*fl/fl*^ *lysm cre* BMDC to stimulate IFN-γ secretion by *p25-tg* T cells (Fig 4L).

Cells derived from *gp130*^*F/F*^ mice, harbouring a mutation that ablates SOCS3 binding to the gp130, show exaggerated gp130-mediated STAT3 responses [28]. Mycobacteria-infected *gp130*^*F/F*^ BMDCs also showed a reduced ability to stimulate IFN-γ secretion *p25-tg* T cells compared to WT cells (Fig 4M).

The frequency of IFN-γ-secreting mycobacteria-specific T cells in lung cell suspensions from *stat3*^*fl/fl*^ *lysm cre* and *stat3*^*fl/fl*^ mice 4 and 8 weeks after infection with *M*. *tuberculosis* was similar. The frequencies of lymphoid cell populations (S2A Fig) and of PPD- and PMA/ ionomycin -stimulated IFN-γ secreting CD4^+^ or CD8^+^ cells (Fig 4N and 4O and S2B and S2C Fig) from lungs *stat3*^*fl/fl*^ *lysm cre* and *stat3*^*fl/fl*^ mice 4 and 8 weeks were also similar. In addition, levels of *ifng*, and the IFN-γ-regulated *inos* and *cxcl9* transcripts were increased in lungs after infection as compared to uninfected controls, but the titers of these transcripts in lungs from *stat3*^*fl/fl*^ *lysm cre* and *stat3*^*fl/fl*^-infected mice were comparable (Fig 4P–4R).

### Myeloid STAT3 hamper T_H_17 responses during *M*. *tuberculosis* infection

**Fig 5:**
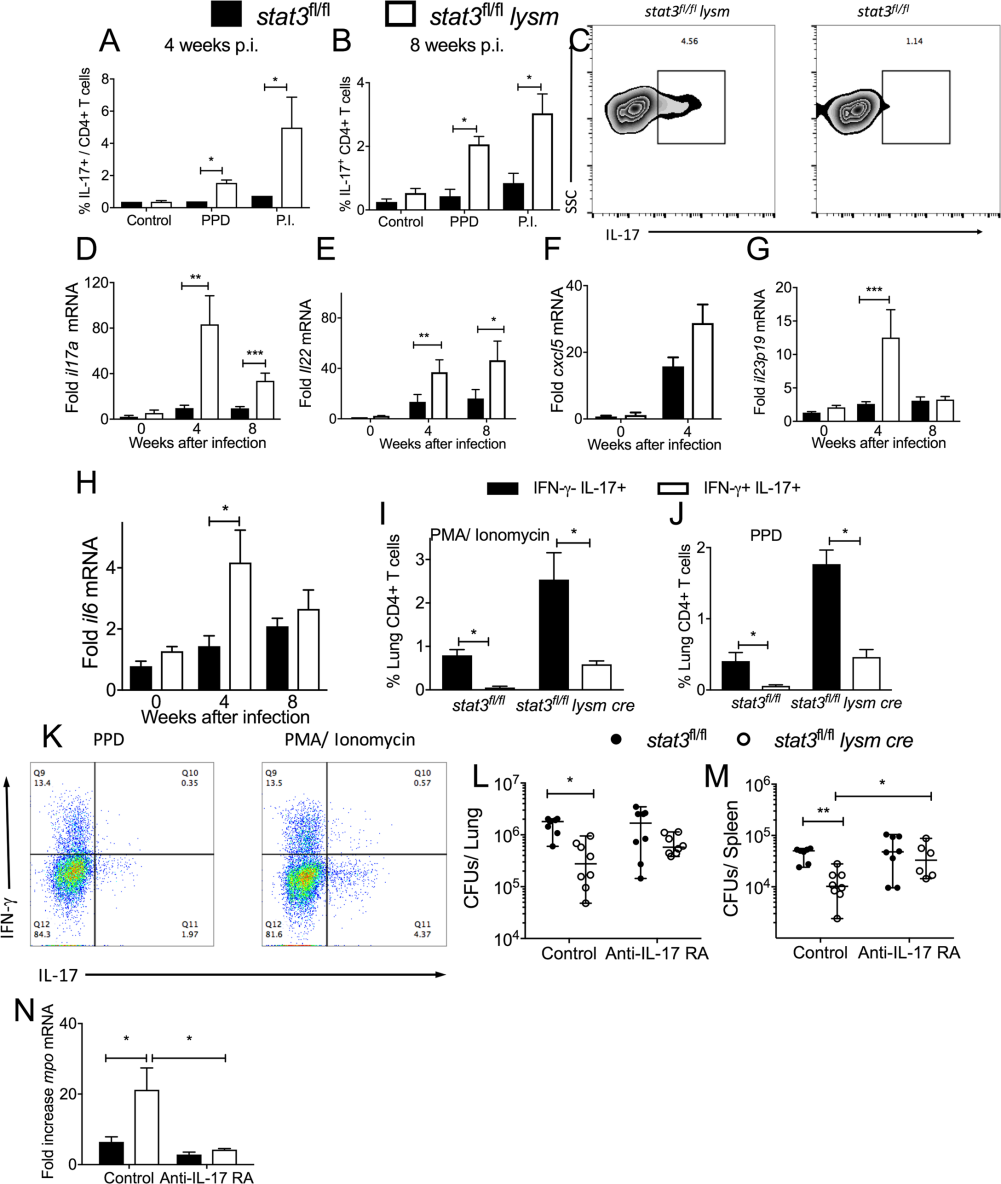
Myeloid cell expression of STAT3 hamper IL-17 secretion by T cells during M. tuberculosis infection. The frequency of IL-17-secreting PPD and PMA and ionomycin (P.I) -stimulated CD4+ pulmonary T cells from *stat3*^*fl/fl*^ *lysm cre* and *stat3*^*fl/fl*^ mice 4 (A) and 8 (B) weeks after infection with *M*. *tuberculosis* was measured by FACS. A representative graph plot from PPD stimulated lungs at 4 w after infection (C) and the mean percentage of IL-17-secreting CD4+ cells ± SEM (A, B) are displayed (n = 6 per group, *p<0.05, **p<0.01 and ***p<0.001 Mann Whitney U test). The mean fold increase of *il17a* (D), *il22* (E), *cxcxl5* (F), *il23p19* (G) and *il6*(H) mRNA ± SEM was measured by real time PCR in the total RNA from lungs of *stat3*^*fl/fl*^ *lysm cre* and *stat3*^*fl/fl*^ mice at different time points after *M*. *tuberculosis* infection (n = 8 per group **p<0.01 Student’s t test). The mean frequency of PPD (I) and PMA and ionomycin (J) -stimulated CD4+ pulmonary T cells from *stat3*^*fl/fl*^ *lysm cre* and *stat3*^*fl/fl*^ mice 8 weeks after infection with *M*. *tuberculosis* co-secreting or not IFN-γ was measured by FACS (n = 4 per group, *p<0.05, **p<0.01 and ***p<0.001 Mann Whitney U test). A representative graph plot from CD3+CD4+ gated PPD and P.I. stimulated IFN-γ and / or IL-17+ lung cells (K) is depicted. The frequency of both IL-17 and IFN-γ and only IL-17+ secreting cells from *stat3*^*fl/fl*^*lysm cre* is higher as compared to *stat3*^*fl/fl*^ controls; the frequency of IL-17+/IFN-γ- is higher than IL-17+/IFN-γ+ cells. This was determined after either PPD or PMA/ ionomycin stimulation (Two-way ANOVA *p<0.05 and *** p<0.0001). *Stat3*^*fl/fl*^ *lysm cre* and *stat3*^*fl/fl*^ littermate controls were treated i.p. 1 day before and once per week after aerosol infection with *M*. *tuberculosis* with 500 μg anti-IL-17RA M751or left untreated. Mice were sacrificed 4 weeks after the infection and colony forming units (CFU) per lung (L) and spleen (M) were assessed. The CFU per organ of individual mice and the median per group at the indicated time points after infection are depicted. Differences in CFU are significant (*p<0.05, **p<0.01 Mann Whitney U test). The mean fold increase of *mpo* mRNA ± SEM was measured by real time PCR in lysates from lungs of *stat3*^*fl/fl*^ *lysm cre* and *stat3*^*fl/fl*^ mice 4 weeks after infection with *M*. *tuberculosis* treated or not with anti-IL-17RA as described above (n≥ 4 per group, *p<0.05 Student’s t test) (N).

The neutrophil density and the levels of neutrophil transcripts were enhanced in the lungs of *M*. *tuberculosis*-infected *stat3*^*fl/fl*^ *lysm cre* as compared to control mice (Fig 1D–1H). IL-17 has been shown to stimulate granulopoiesis via G-CSF production and to induce the expression of CXC chemokines involved in granulocyte recruitment [29]. Thus, we investigated whether the increased neutrophil levels in lungs from *M*. *tuberculosis*-infected *stat3*^*fl/fl*^ *lysm cre* was associated with augmented T_H_17 responses. The frequency of IL-17-secreting, PPD-stimulated CD4^+^ T cells from lungs from *stat3*^*fl/fl*^ *lysm cre* mice 4 and 8 weeks after infection with *M*. *tuberculosis* were elevated when compared to *stat3*^*fl/fl*^ controls (Fig 5A–5C). Instead, the frequency of γδ T cells in lungs and the frequency of IL-17 secreting pulmonary γδ^+^ T cells from WT or *stat3*^*fl/fl*^ *lysm cre* infected mice was similar (S3A–S3C Fig).

In addition, levels of *il17a* and *il22*, transcripts that code for T_H_17 cytokines were higher in lungs from *stat3*^*fl/fl*^ *lysm cre* mice than in those from littermate controls when measured at 4 and 8 weeks after infection (Fig 5D and 5E). Higher levels of il17 mRNA were also observed in *stat3*^*fl/fl*^*lysm cre* mice 14 weeks after infection with *M*. *tuberculosis*, while the increase of *Il22* mRNA did not reach statistical significance (S4A and S4B Fig). CXCL5 is a neutrophil chemotactic protein stimulated by IL-17 [30]. The level of *cxcl5* mRNA was increased in lungs from *M*. *tuberculosis*-infected *stat3*^*fl/fl*^ *lysm cre* mice (Fig 5F).

Substantial ***in vivo*** data support the notion that IL-6 and IL-23 are required at different stages of T_H_17-cell differentiation [31, 32]. Levels of *il6* and *il23* mRNA were elevated in the lungs of *M*. *tuberculosis*-infected *stat3*^*fl/fl*^ *lysm cre* mice when compared to levels in lungs from WT mice (Fig 5G and 5H).

T_H_17 cells show a high degree of developmental flexibility, and when exposed to IL-12 or IL-23, they can rapidly acquire effector functions that are normally associated with T_H_1 responses such as IFN-γ production [33]. These IFN-γ and IL-17 secreting cells were shown to be pathogenic in murine models of autoimmune diseases, and were also associated with murine colitis and human IBDs [34]. The majority of PPD or PMA/ ionomycin-stimulated IL-17 secreting CD4^+^ T cells in lungs from *M*. *tuberculosis-*infected stat3^*fl/fl*^ *lysm cre or stat3*^*fl/fl*^ mice were not IFN-γ co-producers (Fig 5I–5K). We next studied whether IL-17 played a role in the increased control of infection of *stat3*^*fl/fl*^ *lysm cre* mice. For these experiments, mice were treated with neutralizing IL-17RA mab (M751) before and during infection with *M*. *tuberculosis*. Similar bacterial levels were found in lungs and spleens from *stat3*^*fl/fl*^ *lysm cre* and *stat3*^*fl/fl*^ animals treated with anti-IL17RA mAb. As expected, *stat3*^*fl/fl*^ *lysm cre* mice from untreated mice showed reduced bacterial numbers in lungs and spleens than those from *stat3*^*fl/fl*^ controls (Fig 5L and 5M).

The levels of *mpo* mRNA was measured in lungs from anti-IL-17RA to control for the IL17RA neutralization. As expected, levels of *mpo* mRNA were increased in lungs from *M*. *tuberculosis* infected *stat3*^*fl/fl*^ *lysm cre* mice as compared to WT. In contrast, levels of *mpo* mRNA in lungs from infected or anti-IL17RA treated *stat3*^*fl/fl*^ *lysm cre* and *stat3*^*fl/fl*^ mice was similar. Lower titters of *mpo mRNA* were found in *stat3*^*fl/fl*^ *lysm cre* infected mice treated with anti-IL-17RA compared to untreated infected controls, while *mpo* mRNA levels in anti-IL17RA treated or untreated infeceted *stat3*^*fl/fl*^ mice were similar (Fig 5N).

Hence, increased *M*. *tuberculosis* control during infection of *stat3*^*fl/fl*^ *lysm cre* mice is dependent on IL-17, but IL-17 neutralization did not increase the susceptibility to *M*. *tuberculosis* of mice with normal STAT3 function.

### STAT3 expression in antigen presenting cells inhibit the generation of IL-17 secreting mycobacteria-specific T cells

**Fig 6:**
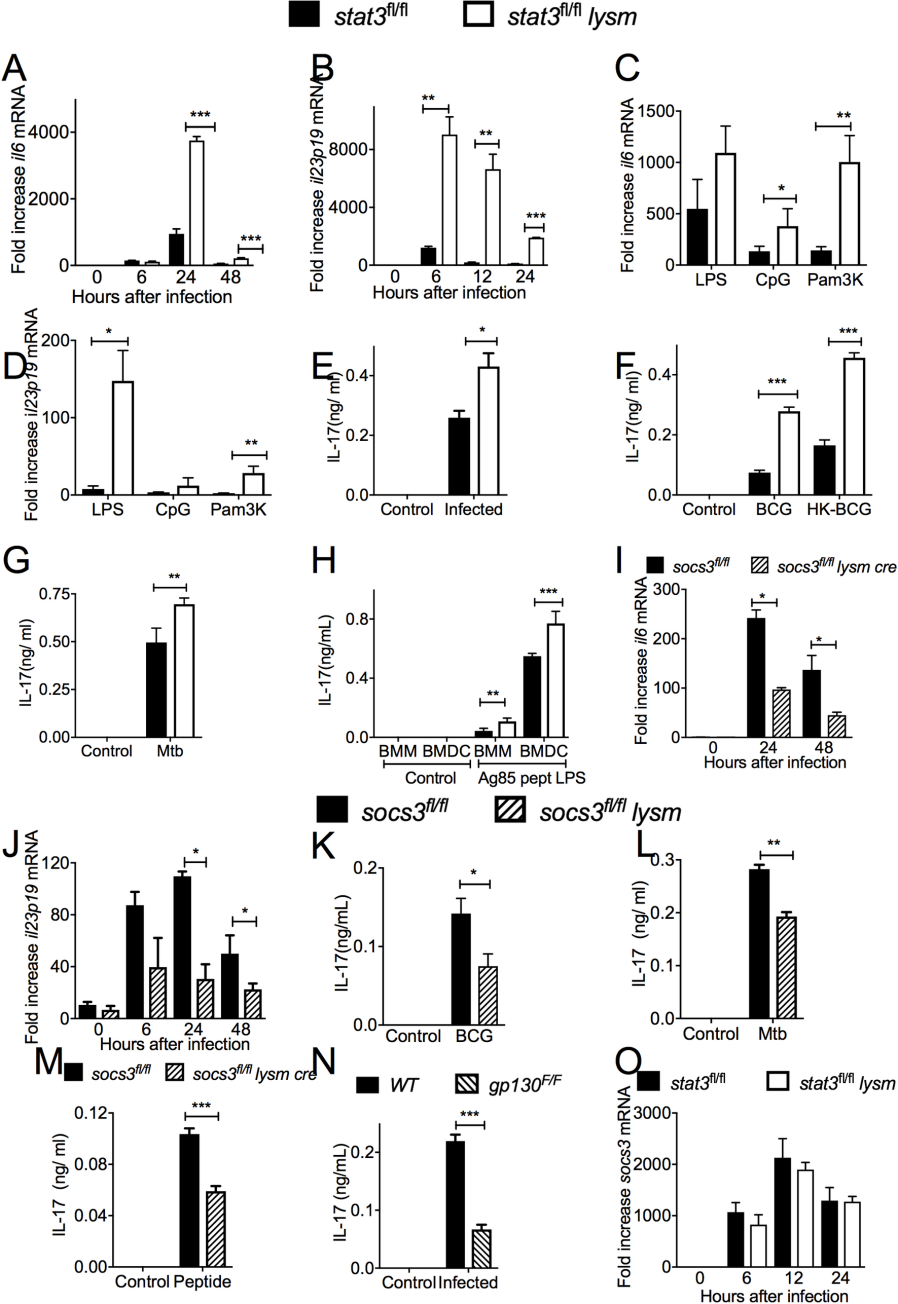
STAT3 and SOCS3 regulate the secretion of IL-17 by antigen-specific T cells. Total RNA was extracted from *stat3*^*fl/fl*^ *lysm cre* and *stat3*^*fl/fl*^ BMDC cultures 24 h after *M*. *tuberculosis* infection. *Il6* and *il23p19* mRNA were measured by real time PCR and normalized to the *hprt* mRNA levels in the same samples. The mean and *il6* (A) *il-23p19* (B) mRNA fold increase ± SEM levels in triplicate independent cultures are depicted (*p<0.05, **p<0.01 and ***p<0.001 Student’s t test). The mean fold increase of *il6* (C) and *il23p19* (D) ± SEM were measured by real-time PCR in triplicate cultures of stat3^*fl/fl*^ *lysm* cre and *stat3*^*fl/fl*^ BMDCs 24 h after stimulation with either LPS, CpG or Pam3K (*p<0.05 and **p<0.01 Student’s t test). *Stat3*^*fl/fl*^ *lysm cre* and *stat3*^*fl/fl*^ BMDC (E-H) or BMM (H) were stimulated with either BCG (E), heat killed BCG (F), *M*. *tuberculosis* (G), or with LPS and the peptide 25 of Ag85b (H) and incubated 6 h after with *p25-tg* CD4+ naïve T cells (at a ratio of 4:1 BMDC). The concentration of IL-17 in the culture supernatants was measured by ELISA 72h after co-incubation. The mean IL-17 ng/ ml ± SEM from triplicate cultures is depicted (* p<0.05; **p<0.01 and ***p<0.001 Student’s t test). Total RNA was extracted from *socs3*^*fl/fl*^ *lysm cre* and *socs3*^*fl/fl*^ BMDC cultures at different times after infection with mycobacteria. The mean *Il6* (I) and *IL23p19* (J) mRNA levels ± SEM levels determined by real time PCR are depicted (**p<0.01 Student’s t test). The concentration of IL-17 was measured 72h supernatants of co cultures of *p25-tg* naïve T cells incubated with either BCG (K), *M*. *tuberculosis* (L) or pept25 and LPS (M)-stimulated socs3^*fl/fl*^ *lysm cre* and socs3^*fl/fl*^ BMDCs. The mean IL-17 ng/ ml ± SEM from triplicate independent cultures were determined by ELISA (*p<0.05; **p<0.01 and ***p<0.001 Student’s t test). IL-17 was measured in 72 h culture supernatants from control or BCG-infected *gp130*^*F/F*^ BMDC and *p25-tg* T cells. The mean IL-17 ng/ ml ± SEM from triplicate cultures from infected or control BMDCs is shown in (N). Differences are significant at ***p<0.001 Student’s t test. The mean fold increase of *socs3* transcript ± SEM in total RNA from *stat3*^*fl/fl*^ *lysm cre* and *stat3*^*fl/fl*^ BMDC at different time points after *M*. *tuberculosis* infection as compared to uninfected controls were measured by real time PCR are depicted (O).

The role by which STAT3 in activated APCs regulates IL-17 secretion by specific T cells was then studied. The mRNA levels of *Il6* and *il23p19* were both increased in BMM and BMDCs co-incubated with mycobacteria **in vitro** (Fig 6A and 6B and S5A Fig). An increased accumulation of *il6* and *il23p19* mRNA was also observed after stimulation with the TLR agonists LPS, CpG or Pam3K of *stat3*^*fl/fl*^ *lysm cre* as compared to *stat3*^*fl/fl*^ BMM at 6 and 24 h after stimulation (Fig 6C and 6D and S5B Fig). This indicates that STAT3-mediated inhibition of the expression of IL-6 and IL-23 is not restricted to mycobacterial infection or stimulation with mycobacterial molecules. Supernatants from cultures of mycobacteria-infected *stat3*^*fl/f*^ *lysm cre* BMM or BMDC co-incubated with naïve *p25-tg* T cells contained higher titers of IL-17 than those using *stat3*^*fl/fl*^ controls (Fig 6E–6G). IL-17 levels were also higher in supernatants from *p25-tg* T cells co-incubated with *stat3*^*fl/fl*^ *lysm cre* BMDC stimulated with either live or heat-killed BCG, *M*. *tuberculosis* or peptide 25 from Ag85b in presence of LPS (Fig 6F–6H).

Whether SOCS3 in mycobacteria-infected APCs also regulated IL-17 secretion by antigen-specific T cells was then measured. We found that mycobacteria-infected *socs3*^*fl/fl*^ *lysm cre* BMM contained lower levels of *il6* and *il23p19* mRNA than their WT counterparts (Fig 6I and 6J). Moreover, *p25-tg* T cells incubated with *socs3*^*fl/fl*^ *lysm cre* BMM stimulated either with BCG, *M*. *tuberculosis* or the cognate p25 peptide secreted lower levels of IL-17 than those stimulated by WT BMDCs (Fig 6K–6M). Similarly, supernatants from co-cultures of *p25-tg* T cells with mycobacteria-infected *gp130*^*F/F*^ BMDC contained higher levels of IL-17 than those using wild type BMDCs (Fig 6N).

We then asked whether STAT3 deficiency regulated the levels of *socs3* mRNA transcripts in mycobacteria-infected macrophages. *M*. *tuberculosis*-infected *stat3*^*fl/fl*^ *lysm cre* and *stat3*^*fl/fl*^ BMMs showed similar levels of *socs3* mRNA (Fig 6O).

### STAT3 and SOCS3 in T cells show a differential regulation of IL-17 and IFN-γ secretion

**Fig 7:**
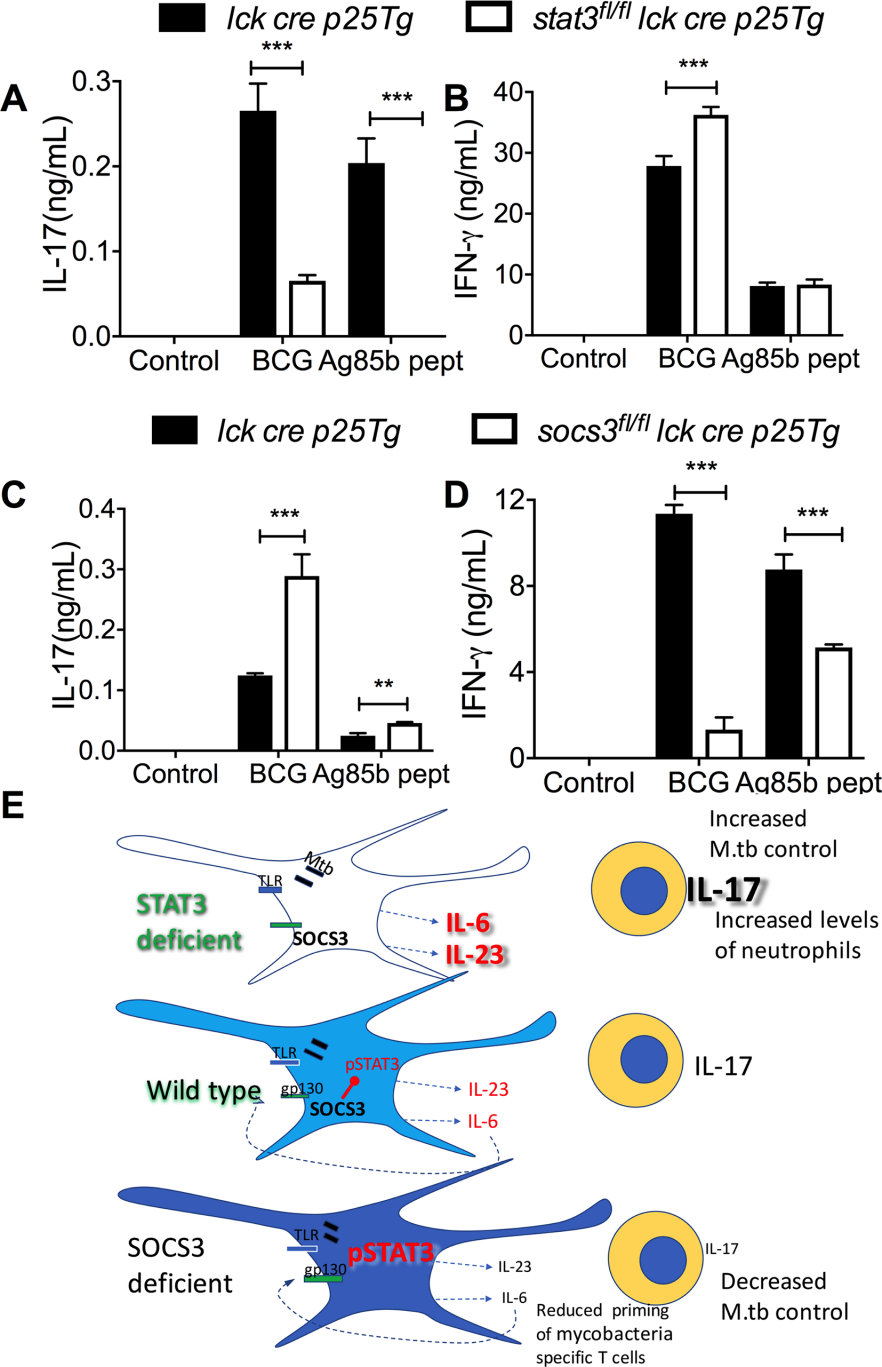
SOCS3 and STAT3 in antigen-specific T cells are important regulators of IL-17 and IFN-γ secretion. *Stat*3^*fl/fl*^ *lck cre p25-tg* T cells (A, B), *socs3*^*fl/fl*^ *lck cre p25-tg* T cells (C, D) or control *p25-tg* T cells were incubated for 3 days with either BCG, peptide 25 and LPS-stimulated or untreated BMDCs. The mean concentration of IL-17 (A, C) and IFN-γ (B, D) ± SEM in supernatants from triplicate cultures is shown (**p<0.01 and ***p<0.001 Student’s t test). *Graphical summary* (E): Mice deficient in STAT3 in myeloid cells show increased resistance while *socs3*^*fl/fl*^ *lysm cre* mice displaying augmented STAT3 activation had impaired resistance against infection with *M*. *tuberculosis*. The differential control of infection in excess or deficiency of STAT3 is not due to the intrinsic regulation of bacterial control by macrophages but rather to differences in the ability of DCs to regulate the differentiation of specific T-cells. *Stat3*^*fl/fl*^ *lysm cre* APCs release higher levels of IL-6 and IL-23 after stimulation with mycobacterial or other TLR agonists, while secretion of these cytokines is reduced in *socs3*^*fl/fl*^ *lysm cre* APCs. *Stat3*^*fl/fl*^ *lysm cre* APCs show improved ability to trigger IL-17 release by mycobacteria-specific T cells while the opposite is observed when using *socs3*^*fl/fl*^ *lysm cre* APCs. The IL-17 secretion by T cells is also controlled via gp130R signalling, indicating an autocrine or paracrine loop by IL-6 family cytokines. The increased resistance to *M*. *tuberculosis* infection of *stat3*^*fl/fl*^ lysm cre mice was IL-17 dependent. *Socs3*^*fl/fl*^ *lysm cr*e (but not *stat3*^*fl/fl*^ *lysm cre)* DCs improved capacity of mycobacteria-specific T cells priming *in vivo*.

Different to the inhibitory role of STAT3 in myeloid cells we here showed, STAT3 expression in T cells has been indicated to be required for T_H_17 cell differentiation **in vitro** and **in vivo** [31]. SOCS3, via hyperactivation of STAT3, has been shown to increase IL-17 secretion [35]. In order to compare the role of STAT3 and SOCS3 in T cells and APCs in the regulation of cytokine secretion by T cells, *stat3*^*fl/fl*^ *lck cre p25-tg* and *socs3* ^*fl/f*^ *lck cre p25-tg* mice were generated. The culture supernatants of *stat3*^*fl/fl*^ *lck cre p25-tg* T cells stimulated with BCG-infected or Ag85b peptide-pulsed BMDCs showed low or undetectable levels of IL-17 as compared to controls (*lck cre p25-tg* T cells) (Fig 7A). Instead IL-17 levels in supernatants from *socs3*^*fl/fl*^ *lck cre p25-tg* T cells co-incubated with BCG or peptide loaded BMDCs were higher than controls (Fig 7C).

The titters of IFN-γ in the supernatants of *stat3* ^*fl/fl*^ *p25-tg* T cells incubated with BCG- (but not with Ag85b peptide-) stimulated BMDCs were higher than those incubated with control T cells (Fig 7B). IFN-γ levels in supernatants from mycobacteria or peptide pulsed BMDCs incubated with *socs3*^*fl/fl*^ *lck cre p25-tg* were instead lower than those using control *p25-tg* T cells (Fig 7D).

Thus, STAT3 in APCs and T cells, has a dissimilar ability to regulate IL-17 secretion by ag-specific T cells, while SOCS3 in APCs and T cells promote T cell mediated IFN-γ secretion.

## Discussion

We report here that stat3^*fl/f*^ *lysm cre* mice show reduced *M*. *tuberculosis* load in lungs and spleens, indicating that STAT3 expression in myeloid cells is detrimental for the control of infection with *M*. *tuberculosis*. Despite reduced area of the lung occupied by granuloma the area with inflammation was not reduced and the numbers of infiltrating pulmonary neutrophils were elevated in stat3^*fl/fl*^*lysm cre* mice. Neutrophil accumulation late during infection have been associated with susceptibility to *M*. *tuberculosis*, whereas early after infection neutrophils play a protective role and contribute to early priming of T cells in the draining lymph node [36–39].

Although mice lacking STAT3 expression in bone marrow progenitors display peripheral neutrophilia under resting conditions [40], the pathways involved in neutrophil mobilization and response to chemokines during inflammation have been shown to be STAT3 dependent [41, 42]. Thus, we consider it unlikely that the increase in pulmonary neutrophils observed during infection occurs as a direct consequence of STAT3 deficiency in these cells. Rather, increased levels of IL-17 and IL-22, cytokines that stimulate the expression of neutrophil recruiting chemokines [29], might contribute to the accumulation of granulocytes in lungs from *M*. *tuberculosis*-infected *stat3*^*fl/fl*^ *lysm cre* mice. In agreement with this hypothesis, the frequency of IL-17 secreting mycobacteria-specific CD4^+^ T cells, but not of γδ^+^ T cells, were elevated in the lungs from *stat3*^*fl/fl*^ *lysm cre* mice compared to controls.

STAT3 expression in APCs proved to be a major regulator of the expression of cytokines that control T cell differentiation. This was shown in STAT3- and in SOCS3-deficient APC, which display higher levels of activated STAT3 when stimulated with mycobacteria or other innate receptor agonists [21, 43]. Thus, while mycobacteria-infected STAT3 deficient APCs showed an improved ability to trigger IL-17 secretion by antigen-specific T cells, the opposite was observed using socs3^*fl/fl*^ *lysm cre* or *gp130*^*F/F*^ BMDCs or macrophages as APCs.

We observed an increased IFN-γ secretion by antigen-specific T cells incubated with STAT3-deficient, mycobacteria-infected APCs. However, IFN-γ responses were not increased **in vivo**. Whether this is due to the present of cytokines that might stimulate IL-17 responses while antagonizing T_H_1 cells (such as for example TGF-β) remains to be explored. Whereas in vitro cultures used provide a proper tool to gain mechanistic insights, the diversity of populations, the tissue localization and the balance between the host immune responses and mycobacteria in the chronic infection might account for differences observed between *in vitro* responses and the control of infection in mice.

The increased resistance to *M*. *tuberculosis* in stat3^*fl/fl*^ *lysm cre* mice is mirrored by data showing that mice with SOCS3 deficiency in myeloid cells display reduced resistance to TB and toxoplasmosis [21, 44]. Here we show that socs3^*fl/fl*^ *cd11c cre* mice. CD11c cre in these mice has been shown to be expressed in ca 90% of splenic DCs, compared with <10% of lymphocytes and <1% of myeloid cells such as granulocytes [25]. Thus, these animals in which SOCS3 is deleted in DCs but not in inflammatory macrophages and neutrophils [25] are also more susceptible to infection with *M*. *tuberculosis*. This supports that the major role of STAT3 and SOCS3 in myeloid cells in the control of infection with *M*. *tuberculosis* is not due to an altered ability of SOCS3 or STAT3-deficient macrophages to control the growth of the intracellular mycobacteria *in vitro*, as shown here and ref [21].

To our knowledge, this is the first report showing that STAT3 deficiency in myeloid cells promotes IL-17 secretion by antigen-specific T cells **in vitro** and **in vivo**. Such a role was related to the increased secretion of T_H_17 inducing IL-6 and IL-23 by STAT3-deficient APCs. The increased expression of IL-6 and IL-23 in stat3^*fl/fl*^ *lysm cre* APCs was not restricted to the infection with attenuated or virulent mycobacteria since, it was observed after incubating mutant APCs with different TLR agonists or bacterial lysates, confirming previous data [45]. The opposite effect was observed using socs3^*fl/fl*^ *lysm cre* BMM, which were poor inducers of IL-17 secretion by mycobacteria-specific T cells. In relation to this, DC that secrete IL-12p40 (required for T cell differentiation into Th17 or Th1) in the lymph nodes of mycobacteria infected mice are primarily uninfected [46].

Since IL-6 can be produced by various hematopoietic and non-hematopoietic cells, we suggest that APCs are relevant cellular sources of IL-6 for the differentiation of IL-17 secreting cells during infection. Moreover, our data using *gp130*^*F/F*^ BMDCs indicate that DC-derived IL-6 acts in an autocrine/ paracrine manner on DCs to regulate their ability to stimulate IL-17 secretion by T cells. A role for gp130/ IL6/ STAT3 pathway in susceptibility to *M*. *tuberculosis* has been previously determined. The high susceptibility of *gp130*^*F/F*^ mice to infection with *M*. *tuberculosis* was not observed in *gp130*^*F/F*^*il6*^-/-^ or *gp130*^*F/F*^ *stat3*^*+/-*^ mice [21].

Our observations on stat3^*fl/fl*^ *lysm cre* mice are reminiscent of those seen in *il10*^-/-^ or anti-IL-10R mAb treated mice that resulted in enhanced lung T_H_1 and T_H_17 responses after BCG vaccination [47]. Depletion of IL-10 resulted in elevated protection to *M*. *tuberculosis* in some studies but not others [47–50]. However different to our model, IL-10 might not only impair the functions of APCs but is also secreted by T cells and has a direct inhibitory effect on T_H_1 or T_H_17 cells [51].

We showed that the improved *M*. *tuberculosis* control in stat3^*fl/fl*^ *lysm cre* is IL-17-mediated, since administration of neutralization anti-IL-17RA antibodies abrogated differences in bacterial burden between mutant and control mice. IL-17 might contribute to long term protection, control of infection after vaccination or control of hypervirulent strains of *M*. *tuberculosis* [52–55]. IL-17 has been suggested to induce of protective T_H_1 responses against mycobacterial infection [52, 56]. IL-17 has been also shown to mediate CXCL13 induction in the lung, a chemokine that contributes to the localization of pro-inflammatory cytokine-producing CXCR5+ T cells within lymphoid structures, promoting those macrophage activation and mycobacterial control [53, 57].

However, other studies have indicated that IL-17 is dispensable after primary infection with *M*. *tuberculosis* [58]. In line with the later observations, we observed similar *M*. *tuberculosis* load in lungs or spleens of WT mice treated or not with anti-IL-17RA.

Levels of MHCII and CD80 and CD86 were lower on socs3^*fl/fl*^ *lysm cre* BMDCs after mycobacterial stimulation confirming previous findings showing reduced MHCII and co-stimulatory molecules after LPS stimulation of SOCS3-deficient BMDCs [59]. Furthermore, the activation of mycobacteria specific *p25-tg* T cells was also diminished in MLN from *M*. *tuberculosis*-infected socs3^*fl/fl*^ *lysm cre* mice as compared to controls. Of importance, p25-tg T cell proliferation was not detectable in the MLN of mice infected with an Ag85b deficient strain of *M*. *tuberculosis* indicating the specificity of p25-tg T cell priming [60]. The Ag85b KO *M*. *tuberculosis* strain grew in the lungs and disseminated to the MLN at a rate equivalent to that of wild-type bacteria. Instead, *stat3*^*fl/fl*^ *lysm cre* and control BMDCs expressed similar levels of MHC-II and co-stimulatory molecules after mycobacterial stimulation in vitro and similar levels of activated antigen-specific T cells in vivo. Different to these results, STAT3 deficient APCs have been shown increased MHCII levels after IL-6 stimulation [61].

Finally, the role of STAT3 in T cells in regulation of antigen-specific IFN-γ and IL-17 T cell responses was investigated. Contrary to the role of STAT3 in APCs, IL-17 secretion was hampered in mycobacteria-specific STAT3-deficient T cells. STAT3 is required for the responses to both IL-6, IL-21 and IL-23 and for the expression of RORγt by T cells [62]. Instead, SOCS3-deficient antigen-specific T cells secreted higher IL-17 levels as previously reported in other systems [35], while IFN-γ responses were inhibited. Thus, while the role of STAT3 in T cells in the control of *M*. *tuberculosis* remains to be studied, these results illustrate the pleiotropic effect of STAT3 in regulation of infection-induced immune responses in different cell types.

In summary, we here showed using SOCS3- and STAT3-deficient mice that STAT3 in myeloid cells is detrimental for the control of infection with *M*. *tuberculosis*. Surprisingly, this occurs via impairing secretion of IL-17 by antigen-specific T cells (Fig 7E).

## Materials and methods

### Ethics statement

The animals were housed and handled at the Dept. of Microbiology, Tumor and Cell Biology and the Astrid Fagreus Laboratory, Karolinska Institute, Stockholm, according to directives and guidelines of the Swedish Board of Agriculture, the Swedish Animal Protection Agency, and the Karolinska Institute (djurskyddslagen 1988:534; djurskyddsförordningen 1988:539; djurskyddsmyndigheten DFS 2004:4). The study was performed under approval of the Stockholm North Ethical Committee on Animal Experiments permit number N397/13 and N487/11. Animals were housed under specific pathogen-free conditions.

### Mice

Mice containing loxP-flanked *stat3* and *socs3* alleles have been described before [43]. For a myeloid-specific deletion these were bred with transgenic *lysm cre* mice [63]. *Socs3*^*fl/fl*^ mice were also bred with *cd11c cre* transgenic animals. *Stat3*^*fl/fl*^ *or socs3*^*fl/fl*^ littermates negative for *cre* expression were used as controls for all experiments. *Gp130*^*F/F*^ mice with a homozygous substitution of tyrosine (Y)_757_ to phenylalanine (F) within the common IL-6 family receptor gp130 abrogating the SOCS3 binding site have been described before [64]. Transgenic T cell receptor *p25-tg* mice with a T-cell receptor specific for peptide 25 (aa 240–254) of mycobacterial Ag85B on H2^b^ haplotype were used [65]. *p25-tg rag2*^-/-^ mice expressing ECFP were generated by crossing *p25-tg* with *rag1*^-/-^ mice [65] with ECFP mice on a *rag2*^*-/-*^ background (kindly provided by Dr. Ronald Germain, NIAID, NIH). The ECFP expression co-localized with Vβ11 used by p25tg T cells [65]. *Socs3*^*fl/fl*^ *lck cre* and *stat3*^*fl/fl*^ *lck cre* mice deficient in SOCS3 and STAT3 in T cells were crossed with *p25-tg* mice to generate *p25-tg socs3*^*fl/fl*^ *lck cre* and *p25-tg stat3*^*fl/fl*^ *lck cre* mice. *P25-tg lck cre* mice were also obtained and used as controls.

### Infection and infectivity assay

BCG Montreal and *M*. *tuberculosis* Harlingen were grown in Middlebrook 7H9 (Difco, Detroit, MI) supplemented with albumin, dextrose, catalase and, for BCG cultures, 50 μg/ ml hygromycin (Sigma, St. Louis, MO). Mice were infected with 250 *M*. *tuberculosis* Harlingen strain by aerosol using a nose-only exposure unit (In-tox Products, Uppsala, Sweden)[66].

Bacteria were quantified on Middlebrook 7H11 agar containing 10% enrichment of oleic acid, albumin, dextrose, catalase, 5 μg of amphotericin B per ml and 8 μg/ ml polymyxin B grown for 3 weeks at 37°C.

### Generation of mouse bone marrow-derived macrophages

Bone marrow was extracted from tibia and femurs of mice and resuspended in DMEM containing glucose and supplemented with 10% FCS and 30% L929 cell-conditioned medium (as a source of macrophage-colony stimulating factor). Bone marrow cells were passed through a 70 μm cell strainer, plated and incubated for 6 days at 37°C, 5% CO_2_. Bone marrow-derived macrophage (BMM) cultures were then washed vigorously to remove non-adherent cells, trypsinized, counted and cultured for one day at 37°C in 24, 12 or 6 well plates. We have previously shown that these BMM are F4/80^+^, CD14^+^ and Mac-3^+^ [67].

### Quantification of intracellular mycobacteria

In order to quantify intracellular *M*. *tuberculosis* uptake and growth, BMM cells were plated on glass slides at 2.10^5^ cells per well in 24 well plates, incubated for 4 h with *M*. *tuberculosis* (MOI 2) and washed with PBS for 3 times to remove the extracellular bacteria before either fixation or replacing the medium. Three days after infection cells were washed with PBS, fixed with 2% PFA and stained with phalloidin to label F-actin (Life technologies, 1:100), DAPI (1:500) and auramine-rhodamine T to label mycobacteria (BD). Micrographs from infected macrophages (400X) were obtained and a total of at least 1000 BMM from 3 independent cultures and categorized as infected or uninfected. The intracellular *M*. *tuberculosis* were enumerated. BMM harboring 5 or more bacteria were considered as containing 5. In some cultures, mycobacterial CFU from BMM 6 days after infection were determined.

### Generation of mouse bone marrow-derived dendritic cells

Mouse bone marrow-derived dendritic cells (BMDC) were differentiated as previously described [68]. Briefly, bone marrow was extracted from tibia and femurs and cell suspensions cultured in RPMI-1640 medium containing 10% FCS and 2 ng/ ml GM-CSF (Peprotech, Rocky Hill, NJ). Fresh medium and cytokine were replaced after 3 days. After six days of culture, loosely adherent cells were harvested and seeded in concentrations for infection. Harvested cells were further selected for CD11c expression with magnetic beads (Miltenyi Biotech) before seeding.

### T cell priming *in vitro*

BMDC or BMM were stimulated with either live or heat killed BCG, *M*. *tuberculosis* or Ag85b peptide in presence of LPS for 6 h. Then, cells were washed and co-incubated with *p25-tg* CD4+ lymph node transgenic T cells from *rag2*^*-/-*^ *p25-Tg* mice (at a ratio of 4:1 BMDC). The cultures were further incubated for 24–48 hs at 37C 5% CO_2_. At these time points the concentration of IFN-γ and IL-17 in the supernatants was measured by ELISA.

### Real time PCR

Transcripts were quantified by real time PCR as previously described[66]. *Hprt* was used as a control gene to calculate the ΔC_t_ values for individual samples. The relative amount of cytokine/ *hprt* transcripts was calculated using the 2^-(ΔΔCt)^ method. These values were then used to calculate the relative expression of cytokine mRNA in uninfected and infected cells and tissues.

### Flow cytometry and intracellular cytokine staining

Lungs were perfused with PBS through the heart before removal from mice. Lungs were mechanically minced into small pieces and digested with 3 mg/ ml Collagenase D and 30 μg/ ml DNase I for 1 h at 37°C, and single-cell suspensions prepared by filtering lung tissue through 70-μm nylon cell strainers. To further remove impurities cells were loaded in 40/ 70% Percoll gradient in PBS and centrifuged 30 min room temperature. The cells at the interphase were collected and washed. Single spleen cell suspensions were obtained by mechanical disruption, lysis of erythrocytes and straining over a 70-μm nylon mesh. Lung, lymph node and spleen cells were stained for CD3, CD4, CD8, γδ-*TCR*, CD62L, CD69, CD44, CD11b, CD11c, Ly6C and Ly6G (all eBioscience) and fixed before acquisition.

For determination of IFN-γ and IL-17-producing cells, lung cells were incubated with PPD or with 50 ng/ml phorbol myristate acetate (PMA) and 2 μg/ml ionomycin (Sigma) for 6 or 18 h at 37 ^o^C. Brefeldin (10 μg/ ml) was added to the cultures the last 4 h of stimulation. Cells were then stained with cell population-specific antibodies, and live/ dead staining, fixed, permeabilized using leukocyte permeabilization reagent IntraPrep™ (Immunotech, Marseille, France) and further stained with anti-IL-17a or anti-IFN-γ (eBioscience).

Data were acquired in a CyAn™ ADP (Beckman Coulter) or an LSRII Flow cytometry and analyzed with FlowJo software (Tree star Inc., Ashland, OR).

### Histopathological analysis

Formalin fixed left lungs of mice experimentally inoculated with *M*. *tuberculosis* were blocked on paraffin. From each lung sample 4 sections were obtained, one longitudinal along the long axis of the lobe and 3 across/transversal of the remaining piece of lung.

The blocks were processed and sections were stained with haematoxylin-eosin. All sections were interpreted by the same pathologist (D. G-W.) and scored semi-quantitatively, blinded to the variables of the experiment.

The following features were scored:

- Lung area occupied with granulomas (% of the total area of the section)
- Lung area free of lesions or area of healthy lung (% of the total area of the section)

### Statistics

The Mann Whitney test for the bacterial CFU load in vivo and of the ICS analysis. For each experiment, 8–10 control and 8–10 mutant mice were infected. We performed separated experiments for 4 and 8 weeks post infection. One of two independent experiments showing similar results is shown.

The analysis of cytokine secretion or mRNA, histopathological scores and frequencies was done using the Student’s t test for unpaired samples. All *in vitro* experiments were performed at least twice. A two-way ANOVA was used to compare the differences in IL-17 secretion between genotypes, as well as between cells that co-secrete IFN-γ or not.

## Supporting information

S1 Fig Frequency and numbers of pulmonary neutrophils in *stat3^fl/fl^ lysm cre* and control mice at 14 weeks after infection with *M. tuberculosis*. The frequency (A) and numbers (B) of CD11b^+^CD11c^-^Ly6C^dim^ Ly6G^+^ neutrophils in lungs stat3^fl/fl^ lysm cre and stat3^fl/fl^ mice at 14 weeks after infection with M. tuberculosis ± SEM are shown (n = 4 mice per group); representative dot plots of neutrophil staining in lungs are shown (C).
(TIFF)

S2 Fig Frequency of PMA/ionomycin-stimulated IFN-γ-secreting CD4+ and CD8+ T cells from lungs of *stat3^fl/fl^ lysm cre* and *stat3^fl/fl^* mice 4 and 8 weeks after infection with *M. tuberculosis*. The mean frequency of PMA/ ionomycin-stimulated IFN-**γ** secreting CD4+ and CD8+ lung T cells from *stat3*^*fl/fl*^ *lysm cre* and *stat3*^*fl/fl*^ mice at 4 (B) and 8 (C) weeks after infection with *M*. *tuberculosis* ± SEM was measured by FACS (n = 4 per group).
(TIFF)

S3 Fig Frequency of pulmonary lymphoid cell populations in stat3fl/fl lysm cre and stat3fl/fl mice after infection with M. tuberculosis. The mean frequency of CD4^+^, CD8^+^ and **γδ**^+^ cells within lung CD3+ T cells from *stat3*^*fl/fl*^ *lysm cre* and *stat3*^*fl/fl*^ mice 8 weeks after infection with *M*. *tuberculosis* was measured by FACS (n = 4 per group) (A).
The mean frequency of PPD and PMA/ ionomycin-stimulated IL-17 secreting **γδ**^+^ pulmonary T cells from *stat3*^*fl/fl*^ *lysm cre* and *stat3*^*fl/fl*^ mice at 4 (B) and 8 (C) weeks after infection with *M*. *tuberculosis* was measured by FACS (n = 4 per group).
(TIFF)

S4 Fig *Il17* and *il22* mRNA accumulation in lungs from *stat3^fl/fl^ lysm cre* and control mice before and 14 weeks after infection with *M. tuberculosis*. The mean fold increase of il17a (A), il22 (B) mRNA ± SEM was measured by real time PCR in the total RNA from lungs of *stat3*^*fl/fl*^ *lysm cre* and *stat3*^*fl/fl*^ mice at 14 weeks after *M*. *tuberculosis* infection (n = 5 per group *p<0.05 Student’s t test).
(TIFF)

S5 Fig Levels of il6 and il23p19 mRNA in *stat3^fl/fl^ lysm cre* and *stat3^fl/fl^* after stimulation with different TLR agonists. The mean fold increase of *il6* (A) and *il23p19* (B) ± SEM were measured by real-time PCR in triplicate cultures of stat3^*fl/fl*^ *lysm* cre and *stat3*^*fl/fl*^ BMDCs 6 h after stimulation with either LPS, CpG or Pam3K (*p<0.05 and ***p<0.001 Student t test).
(TIFF)
