# Supplementary figures and images for: STAT3 expression by myeloid cells is detrimental for the T- cell-mediated control of infection with Mycobacterium tuberculosis
Source: PLoS Pathog. 2018 Jan 16;14(1):e1006809. doi: 10.1371/journal.ppat.1006809 (PMC5800682; doi:10.1371/journal.ppat.1006809)

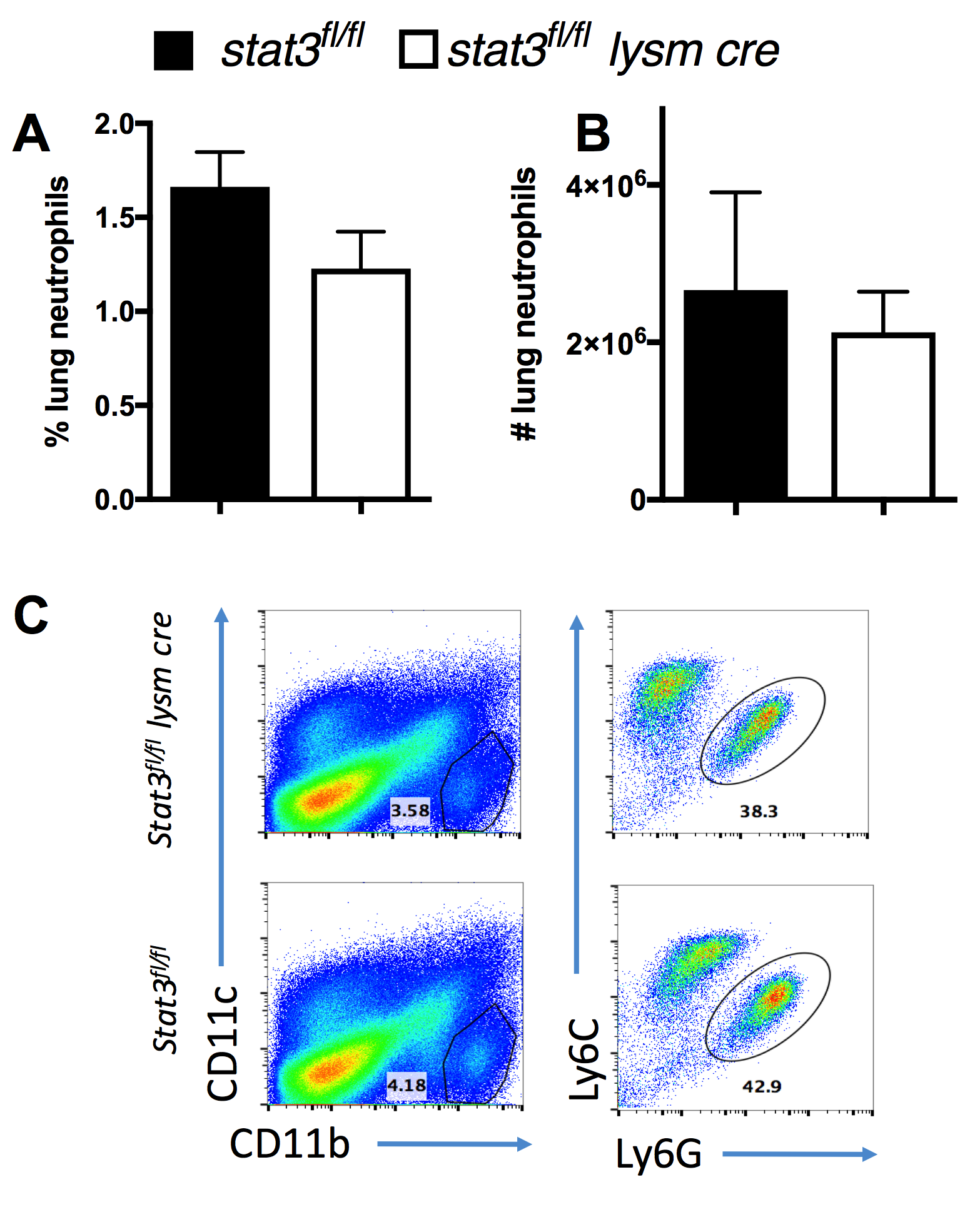

Supplement: S1 Fig — The frequency (A) and numbers (B) of CD11b+CD11c-Ly6Cdim Ly6G+ neutrophils in lungs stat3fl/fl lysm cre and stat3fl/fl mice at 14 weeks after infection with M. tuberculosis ± SEM are shown (n = 4 mice per group); representative dot plots of neutrophil staining in lungs are shown (C). (TIFF) [file ppat.1006809.s001.tiff]

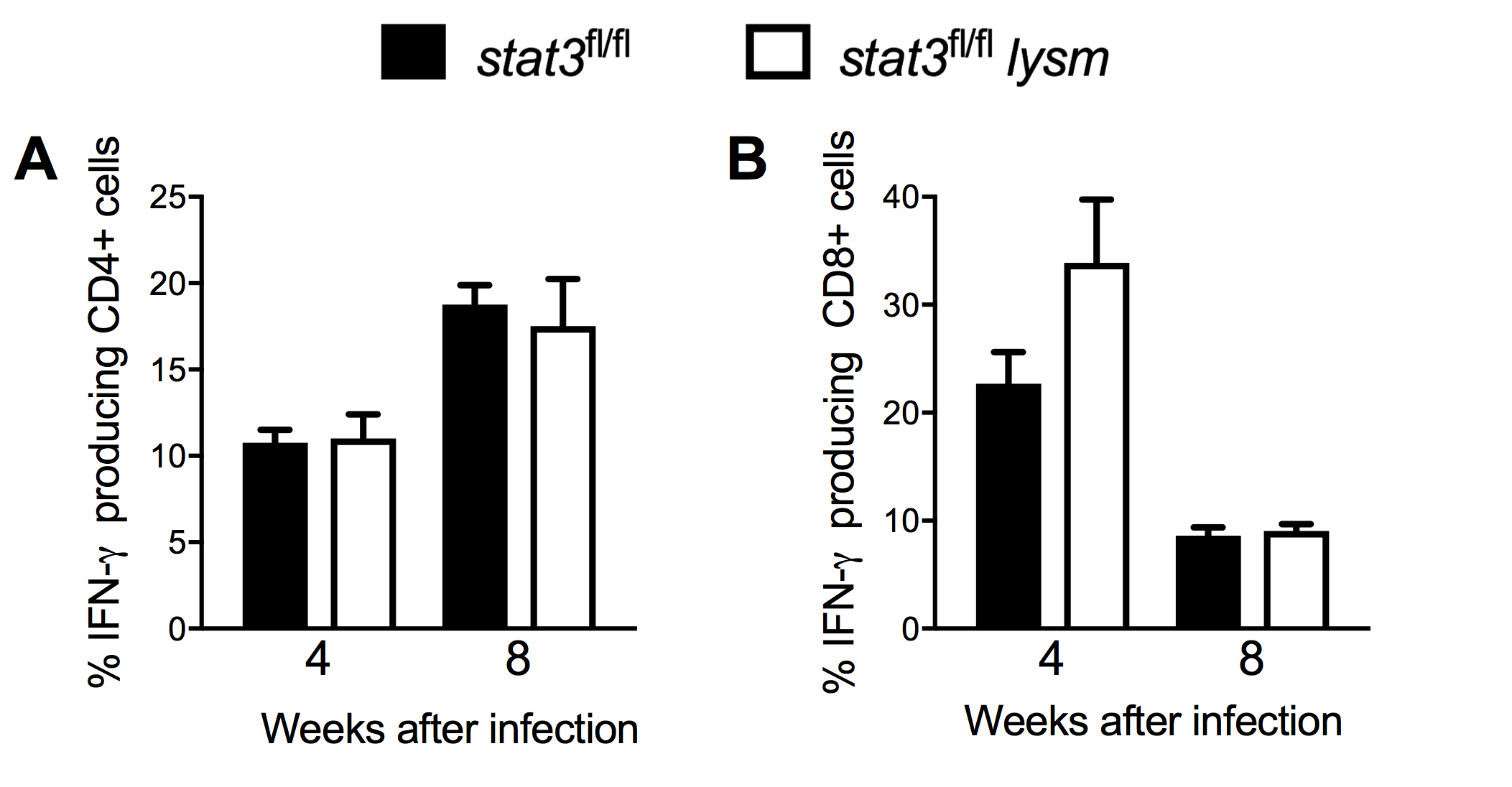

Supplement: S2 Fig — The mean frequency of PMA/ ionomycin-stimulated IFN-γ secreting CD4+ and CD8+ lung T cells from stat3fl/fl lysm cre and stat3fl/fl mice at 4 (B) and 8 (C) weeks after infection with M. tuberculosis ± SEM was measured by FACS (n = 4 per group). (TIFF) [file ppat.1006809.s002.tiff]

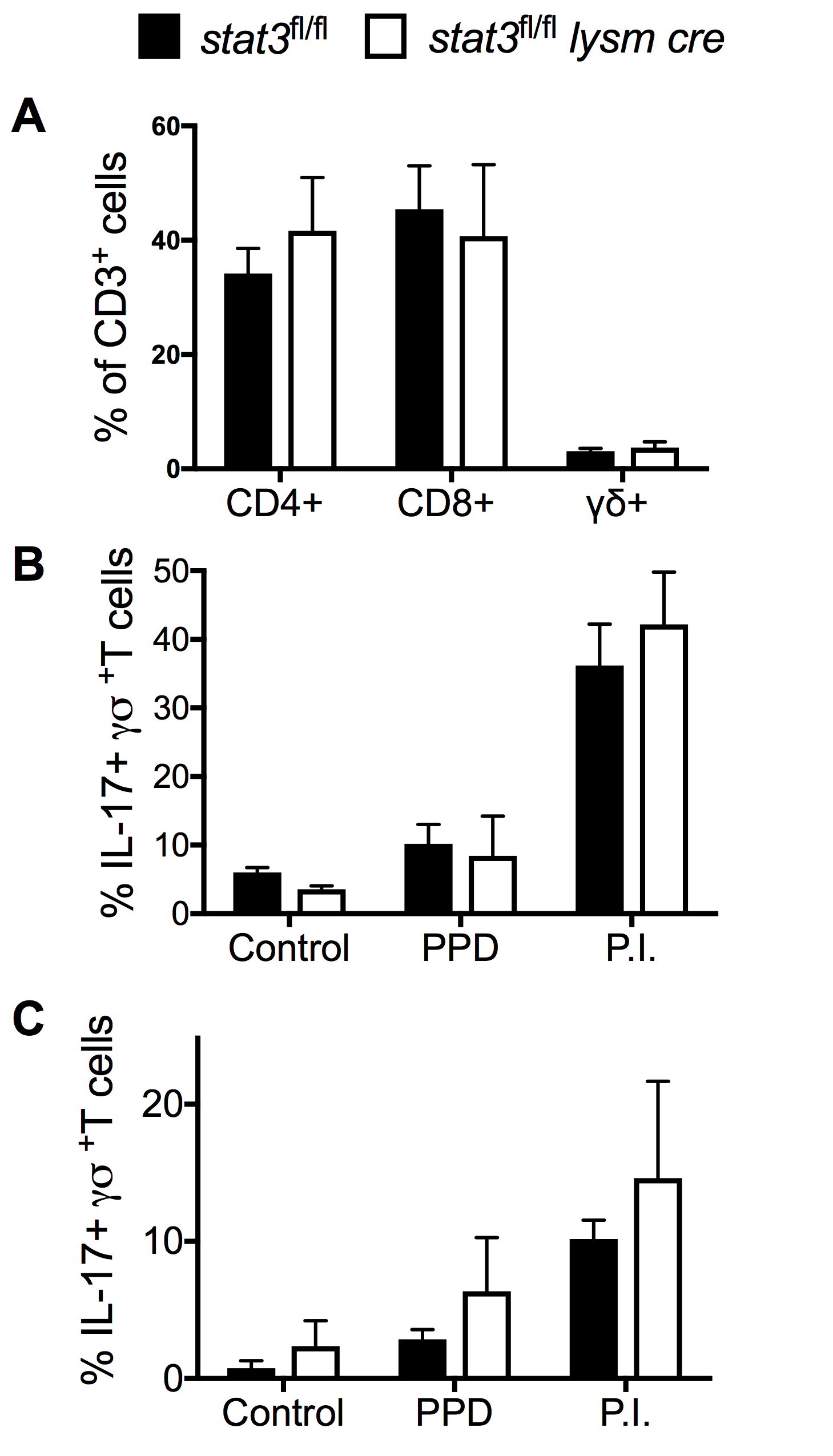

Supplement: S3 Fig — The mean frequency of CD4+, CD8+ and γδ+ cells within lung CD3+ T cells from stat3fl/fl lysm cre and stat3fl/fl mice 8 weeks after infection with M. tuberculosis was measured by FACS (n = 4 per group) (A). The mean frequency of PPD and PMA/ ionomycin-stimulated IL-17 secreting γδ+ pulmonary T cells from stat3fl/fl lysm cre and stat3fl/fl mice at 4 (B) and 8 (C) weeks after infection with M. tuberculosis was measured by FACS (n = 4 per group). (TIFF) [file ppat.1006809.s003.tiff]

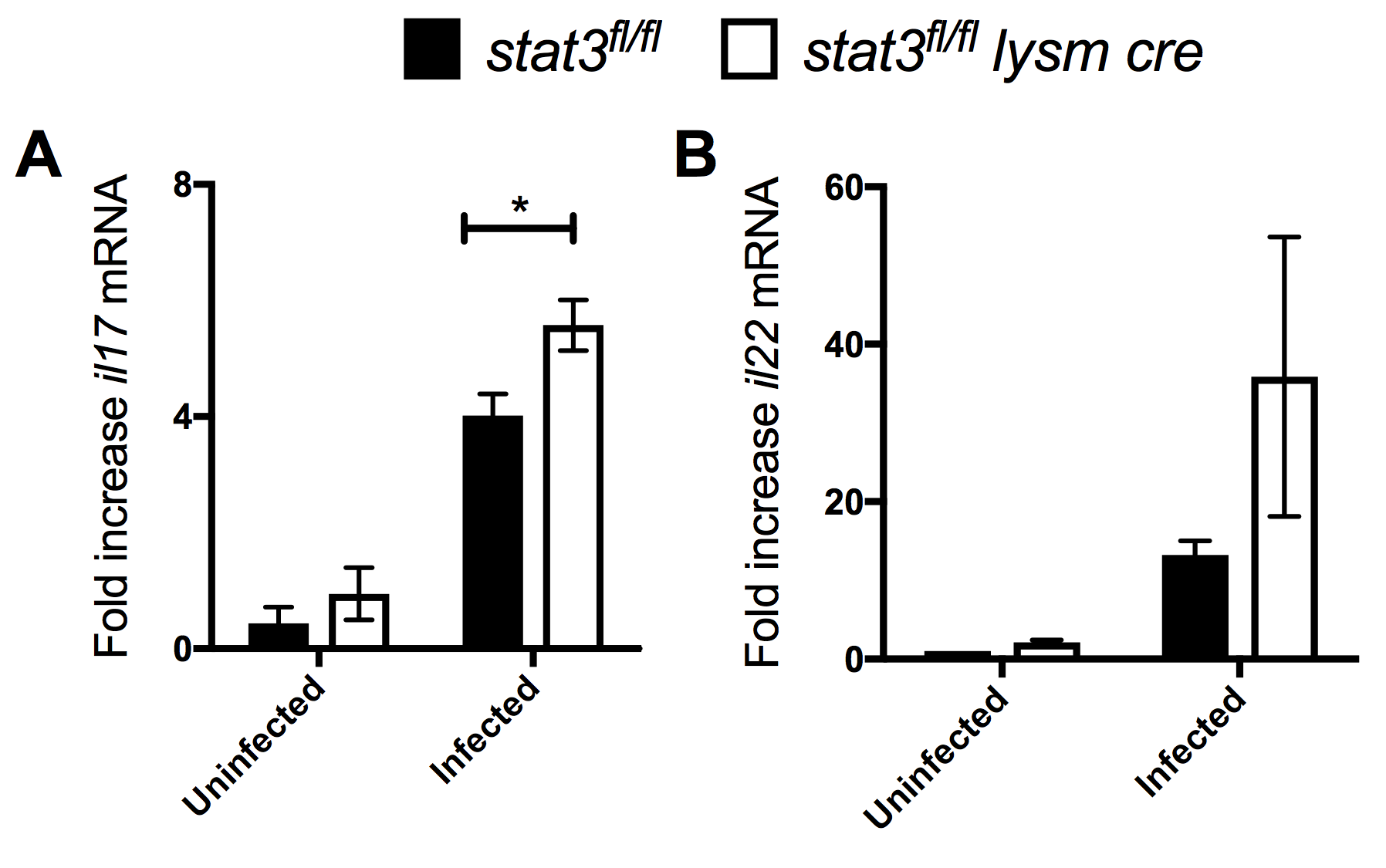

Supplement: S4 Fig — The mean fold increase of il17a (A), il22 (B) mRNA ± SEM was measured by real time PCR in the total RNA from lungs of stat3fl/fl lysm cre and stat3fl/fl mice at 14 weeks after M. tuberculosis infection (n = 5 per group *p<0.05 Student’s t test). (TIFF) [file ppat.1006809.s004.tiff]

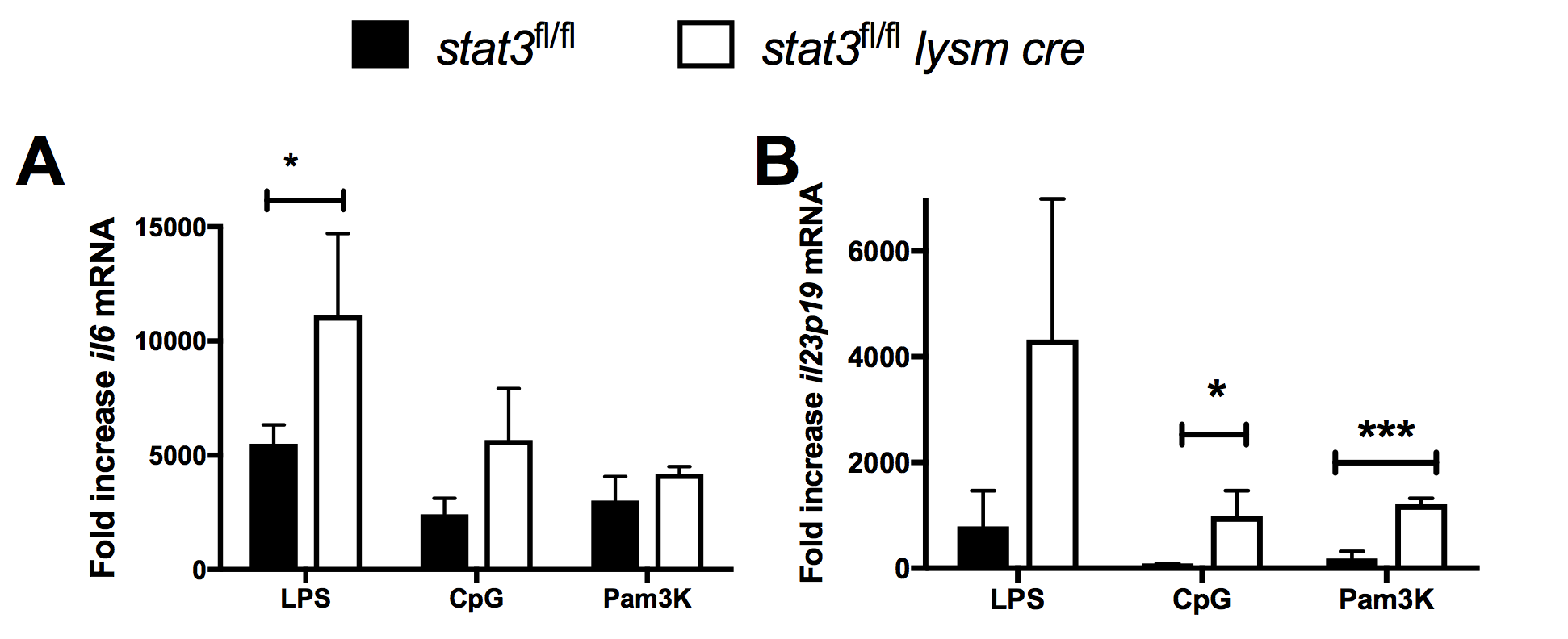

Supplement: S5 Fig — The mean fold increase of il6 (A) and il23p19 (B) ± SEM were measured by real-time PCR in triplicate cultures of stat3fl/fl lysm cre and stat3fl/fl BMDCs 6 h after stimulation with either LPS, CpG or Pam3K (*p<0.05 and ***p<0.001 Student t test). (TIFF) [file ppat.1006809.s005.tiff]
